# Interventions to improve the quality of low back pain care in emergency departments: a systematic review and meta-analysis

**DOI:** 10.1007/s11739-024-03736-y

**Published:** 2024-09-09

**Authors:** Pippa Flanagan, Robert Waller, Ivan Lin, Karen Richards, Piers Truter, Gustavo C. Machado, Vinicius Cavalheri

**Affiliations:** 1 Faculty of Health Sciences, Curtin School of Allied Health, Curtin University, Bentley, WA Australia; 2 Department of Physiotherapy, Fiona Stanley Hospital, Murdoch, WA Australia; 3 Department of Physiotherapy, Rockingham Hospital, Coolongup, WA Australia; 4 Western Australian Centre for Rural Health, The University of Western Australia, Geraldton, WA Australia; 5 School of Health Sciences and Physiotherapy, The University of Notre Dame, Fremantle, WA Australia; 6 Institute for Musculoskeletal Health, The University of Sydney and Sydney Local Health District, Sydney, NSW Australia; 7 Allied Health, South Metropolitan Health Service, Perth, WA Australia; 8 Curtin School of Allied Health, Curtin University, GPO Box U1987, Perth, WA 6102 Australia

**Keywords:** Low back pain, Emergency department, Implementation, Quality of care

## Abstract

Low back pain (LBP) is a common reason people visit Emergency Departments (ED). However, the care provided is often not aligned with guideline recommendations. Despite increasing research aiming to promote guideline-based care in EDs, interventions to best implement recommendations are unknown. This study aimed to identify ED LBP implementation interventions that have been trialed and evaluate their effects on ED-relevant outcomes. A systematic review and meta-analysis, including studies that evaluated interventions to improve the quality of care provided to adults presenting to ED with LBP. Databases searched until May 2023 were Cochrane Library, CINAHL, EMBASE (via OVID), and PEDro. Interventions were categorized according to whether they had a patient, clinician, health service, or multiple-level focus. Where possible, meta-analysis was undertaken. Certainty around the results was assessed using the GRADE criteria. Twenty-eight studies were included. Interventions were categorized as patient (*n* = 2), clinician (*n* = 8), health service (*n* = 12), or multiple-level (*n* = 6) targeted. Overall, interventions successfully reduced the likelihood of receiving an opioid in ED (OR 0.65; 95% CI 0.55–0.75). However, no significant effect on lumbar imaging was demonstrated (OR 0.85; 95% CI 0.64–1.12). Subgroup analyses showed that studies reporting high baseline imaging rates ≥ 36% and those that included systems-based changes significantly reduced imaging (OR 0.60; 95% CI 0.39–0.93; and OR 0.65; 95% CI 0.45–0.94, respectively). A small reduction in ED length of stay was observed in the group exposed to the LBP interventions (mean difference − 0.38 h; 95% CI − 0.58 to − 0.17). Overall, certainty of evidence was deemed low to very low. Interventions were mostly single-system focused with a preference for education-based implementation strategies targeting patients or clinicians. The interventions reduced the use of opioid medication for LBP in ED, but the effects on lumbar imaging rates were uncertain. Further high-quality research is needed to improve LBP care in this setting.

**Supplementary Information:**

The online version contains supplementary material available at 10.1007/s11739-024-03736-y.

## Introduction

People with low back pain (LBP) frequently seek care in Emergency Departments (EDs). In Australian EDs, LBP remains one of the five most common principal diagnoses for people aged between 35 and 64 years [1]. This trend is similar internationally, with a pooled prevalence of LBP in ED estimated at 4.4% [2]. Most of these people will have a benign musculoskeletal cause to their LBP without specific or serious underlying pathology [3]. As such, LBP guidelines consistently recommend avoiding imaging and potentially harmful medicines such as opioids [4–9]. Despite this, over one-third of people in ED receive imaging [10], and opioids are administered at rates of up to 77% [11, 12].

The challenges in providing guideline-based care for people with LBP in ED are often driven by environment-specific factors at the patient, clinician, and health service level. These include patients presenting with severe pain, dysfunction, and specific expectations relating to ED care [13–17], clinician fear of missing serious pathology, the conviction of a need to provide “something” for their patient [13, 14, 18–20], and frequently rotating junior medical staff [14, 18, 19, 21, 22]. Health service factors include competing pressures from limited ED bed capacity and other ED patients [15]. Such environmental pressures may deter clinicians from adopting guideline recommendations that are perceived to be more time-consuming, including patient education and reassurance [23]. These challenges suggest that implementation strategies are needed to support clinicians adopt guideline recommendations into ED practice.

One previous review has identified and examined implementation interventions for LBP in ED [24]. This review included interventions aiming to decrease lumbar imaging in ED with only five studies identified and conflicting evidence demonstrated. Since this review, additional studies have been published targeting other important aspects of ED LBP care including opioid prescribing and self-management education [14]. In addition, the recent release of clinical care standards for LBP in Australia [5] provides a new benchmark to evaluate the effect of implementation strategies to improve LBP care in ED. As such, it is timely to review the recent evidence to evaluate the effects of the strategies that have been trialed.

This systematic review therefore aimed to address the following research questions: (1) What patient, clinician, and health service-targeted interventions implemented in the ED setting to improve the quality of care for patients with LBP have been studied? and (2) What are the effects of such interventions on patient, quality of care, and health-service outcomes?

## Methods

### Study design

This is a systematic review and meta-analysis of studies reporting on interventions implemented in the ED setting that aimed to improve the quality of care for adult patients with LBP. The review was prospectively registered in PROSPERO (CRD42022319295) and was conducted and reported according to the Preferred Reporting Items for Systematic Reviews and Meta-Analyses (PRISMA) 2020 statement [25].

### Eligibility criteria and outcomes

The population of interest was adults (≥ 18 years) presenting to an ED with LBP (including LBP-related leg symptoms and LBP from underlying serious or specific spinal pathology). Original studies of any design that evaluated interventions aiming to improve the quality of ED LBP care were included. Quality of LBP care was defined using the standards of appropriate care described in the 2023 Australian Commission on Safety and Quality in Health Care (ACSQHC) Low Back Pain Clinical Care Standard [5]. The primary outcome of interest was any variable measuring the quality of LBP care within the ACSQHC domains (e.g., the proportion of participants who received lumbar imaging or opioids). Secondary outcomes included patient (e.g., satisfaction), health service (e.g., ED length of stay (LOS)), or other implementation-specific outcomes (e.g., fidelity). Studies were excluded if they: (i) were trials designed to investigate clinical efficacy of interventions; (ii) tested the validity of tools to predict long-term LBP disability if they did not evaluate the implementation of the tool within the ED setting; (iii) only targeted patients presenting to ED with LBP from major trauma; and (iv) included a broad population with musculoskeletal presentations to ED where LBP was not at least 75% of the total cohort of the study or LBP data was not reported separately.

### Search strategy

The search strategy was developed with the assistance of professional Librarians and piloted on the PubMed database including keywords “Emergency Service” or synonyms (e.g., acute care) AND “low back pain” OR synonyms (e.g., spinal pain, lumbar pain) (see online resource; Table S1). The PubMed search was adapted and applied to other databases including the Cochrane Library, CINAHL, EMBASE (via OVID), and PEDro. There was no date limit applied to the search strategy. Only English language texts were included. In addition, a hand search was performed by screening reference lists of included studies and previous review articles. Databases were searched to 30 May 2023.

### Screening and selection

References generated from the database and hand searches were exported to Covidence [26], where duplicates were removed and results were collated for screening. All titles and abstracts underwent independent screening by two review authors (PF and PT), with conflicts resolved by discussion. Full-text articles were further screened against the eligibility criteria by the same two review authors independently, with disagreements resolved by consensus discussion or by consultation with the other review authors (RW, IL, KR, and VC).

### Data extraction

Data from included studies were extracted onto pre-developed forms across four domains: study, participant (including LBP sample characteristics), intervention characteristics (including implementation strategies) and results from individual studies, including details of the comparator groups. Data were extracted by the lead author and checked for accuracy by the other review authors (RW, IL, KR and VC). Contact with five study authors was attempted due to missing or unclear data, with only one response received and no additional data for analysis provided.

### Risk of bias

The Cochrane Risk of Bias tool was used to assess the risk of bias of randomized controlled trials (RCTs) [27]. The risk of bias of non-RCTs was assessed using the Downs and Black checklist [28]. In comparison to other quality rating instruments for observational studies, the Downs and Black checklist has been demonstrated to provide good reliability and validity data in studies of musculoskeletal outcomes [29]. The checklist includes 27 items over five domains (i.e., reporting, external validity, study bias, confounding, and selection bias) to provide a maximum final score of 30. Items not relevant for a specific study design were removed with the total score and denominator scaled accordingly. To allow for comparison of risk of bias, RCTs were also assessed against the Downs and Black checklist. The risk of bias of each study was independently assessed by two review authors (PF and RW, IL, KR, PT, or VC) with conflicts resolved with discussion or taken to the remaining review authors for final consensus. Final Downs and Black score ranges were given risk of bias levels based on previously reported corresponding quality levels [30]: low (≥ 70%), moderate (51–69%), and high (≤ 50%) [30].

### Data synthesis and analysis

To address research question 1, a pragmatic approach was taken to broadly categorize the interventions according to the health system level they predominantly aimed to target (patient, clinician, health service, or multiple levels). Individual implementation strategies used within each intervention were further categorized according to nine different cluster labels from an existing implementation taxonomy (Adapt and tailor to the context, Change infrastructure, Develop stakeholder interrelationships, Engage consumers, Provide interactive assistance, Support clinicians, Train and educate stakeholders, Use financial strategies, Use evaluative and iterative strategies) [31, 32]. An additional category was included and labeled “systems-based” strategies to categorize strategies embedded into existing clinical processes or systems, reducing the reliance on clinician active behavior change (e.g., changes in order sets or clinical documentation requirements) [33].

To address research question 2, random-effects meta-analyses were conducted to generate the pooled effect of interventions on similar outcomes. For dichotomous outcomes, the effect size estimate was odds ratios (OR) with their 95% CIs and the mean difference (MD) with their 95% CIs were used for continuous outcomes. The *I*^2^ statistics was used to calculate the proportion of variance in observed effects that reflect variance in true effects rather than sampling error. The distribution of true effects was explored with the calculation of prediction intervals [34]. Randomized and non-randomized studies were combined in meta-analysis due to insufficient numbers of RCTs in this area. For meta-analysis of ten studies or greater [35], potential sources of clinical or methodological heterogeneity were explored through mixed-effects subgroup analyses. Moderator variables for subgrouping included: health system level targeted by the intervention (patient, clinician, health service or multiple levels); baseline imaging rates (low =  ≤ 35.6%, high =  ≥ 35.7% [10]); risk of bias (low, moderate or high); LBP case definition (if sample included LBP-related leg symptoms and/or serious spinal pathology); whether the populations were identified from presenting complaint (triage) or from ED discharge; and whether the interventions included a systems-based strategy. We chose to explore the influence of systems-based strategies specifically as we feel changes to the systems in which LBP care is delivered may be important to affect change. The *Q*-test was used to evaluate variation in effect sizes between subgroups. The criterion alpha for the *Q*-test was set at 0.10 with *p*-values of < 0.10 demonstrating a significant difference in effect sizes between groups. The five domains of the GRADE assessment (i.e., risk of bias, inconsistency, indirectness, imprecision, and publication bias) [36] were used (via GRADEpro software) to report the certainty around the results for each outcome measure. Where pooling was not possible, a narrative summary was used.

## Results

**Fig. 1:**
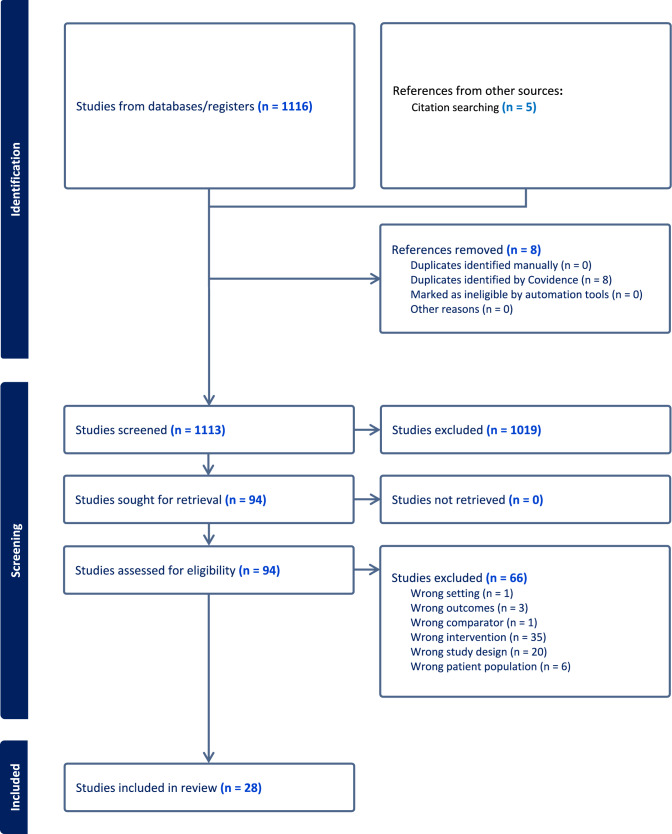
Flow diagram of references identified, excluded, and included in review

The database searches identified 1116 records (Fig. 1). After duplicates were removed, and title/abstract and eligibility screening undertaken, 25 studies remained and were included in the review, with a further three studies identified and added from citation tracking also included (Fig. 1).

### Study characteristics

Table 1 presents an overview of the included studies with a detailed summary in the online resource; Table S2. Studies were published between 1987 and 2022 with most published in the last 10 years (*n* = 22). The studies included EDs that were mostly based in the USA (*n* = 14) and Australia (*n* = 6). Most of the 28 included studies were observational design (*n* = 22). Eleven of these were before-after studies [19, 21, 22, 37–44], eight cohort studies [20, 45–51], and three used interrupted time series design [52–54]. Three included studies were RCTs [14, 55, 56], including one cluster-randomized trial [14]. Three further studies were non-randomized, controlled experimental studies [17, 57, 58].

**Table 1:** Characteristics of included studies

Study and design	Intervention and comparison	Outcome measures	Results	Study findings (Downs and Black score)
*Studies evaluating patient-targeted interventions*				
Chou 2021 [58] USA Controlled cohort study	IG (*n* = NR): employer-mandated switch from low to high deductible health plan (increased patient out-of-pocket expense to access ED imaging) CG (*n* = NR): maintained low deductible health plan	**Quality of care:** Low-value imaging	Relative difference (%): − 5.2; 95% CI − 16.6 to 6.1 *p* = 0.37	Uncertainty whether high deductible health plans reduce low-value imaging (Downs and Black = 80%, low risk of bias)
Sharma 2021 [17] Australia Controlled interrupted time series study	IG (*n* = 99): communication strategy displayed on LCD screens in ED waiting room showing potential harms of unnecessary imaging for LBP plus provision of information leaflet CG (*n* = 238): usual waiting room LCD display plus leaflet accessible by patient	**Quality of care:** LBP presentations with at least 1 imaging test	25% IG v 29% CG OR 0.83; 95% CI = 0.49 to 1.41	Uncertainty whether a waiting room communication strategy reduces lumbar imaging rates (Downs and Black = 73%, low risk of bias)
*Studies evaluating clinician-targeted interventions*				
Bailey 2013 [51] USA Retrospective cohort study	IG (*n* = 100): electronic health information exchange accessed by ED staff during a subsequent ED visit CG (*n* = 700): did not have their electronic health information exchange accessed in ED	**Quality of care:** Repeated lumbar or thoracic diagnostic imaging (radiograph, CT, or MRI)	Repeated imaging: 10% IG v 24.1% CG OR 0.36; 95% CI 0.18 to 0.71	Reduced odds of repeated lumbar imaging in participants that had their health information exchange accessed by staff during their ED visit (Downs and Black = 72%, low risk of bias)
		**Cost:** Total patient-visit estimated cost	$189 IG v $189 CG No difference	
Berezin 2020 [38] Canada Retrospective cohort study	IG (*n* = 515): after national release of Choosing Wisely Canada imaging guidelines CG (*n* = 545): before release of Choosing Wisely Canada imaging guidelines	**Quality of care:** Proportion of spinal imaging	7.6% IG v 8.3% CG OR 0.91; 95% CI 0.58 to 1.42	Uncertainty whether passive dissemination of national imaging guidelines reduce spinal imaging (Downs and Black = 60%, moderate risk of bias)
Blokzijl 2022 [53] Australia Before-after study using time series analysis	IG (*n* = NR): after the statewide release of New South Wales Agency for Clinical Innovation (ACI) acute LBP model of care CG (*n* = NR): before release of ACI model of care (usual care)	**Quality of care:** Proportion of spinal imaging	33.5% IG v 30.4% CG *p* = 0.37	Interrupted time series data did not detect any significant differences in the level or slope of the trend in imaging following the release of the statewide model of care (Downs and Black = 68%, moderate risk of bias)
Chandra 2019 [39] Canada Before-after study	IG (*n* = 672): immediately after the implementation of a LBP knowledge-translation initiative that focused on educating ED clinicians on the Choosing Wisely Canada imaging guidelines CG (*n* = 781): 2 years before implementation of the LBP knowledge-translation initiative (usual care)	**Quality of care:** Frequency of imaging	16.2% IG v 12% CG OR 1.4; 95% CI 1.04 to 1.89	The knowledge-translation intervention was associated with a subsequent increase in the rate of imaging for LBP (Downs and Black = 57%, moderate risk of bias)
Coombs 2021 [14] Australia Stepped-wedge, cluster RCT	IG (*n* = 1392): multifaceted intervention to implement a new LBP model of care in ED that included clinician education sessions and materials, non-opioid pain management options, fast-track referrals to outpatient services, and audit and feedback to staff CG (*n* = 3233): cluster sites in control conditions (usual care) before implementation of intervention	**Patient:** ED patient experiences with care survey-item 31	MD 0.16; 95% CI − 0.72 to 1.03	The multifaceted, clinician-targeted intervention improved clinicians’ beliefs and knowledge regarding LBP and its management and reduced opioid use by 12.3% absolute reduction No effect was seen on other healthcare utilization outcomes including uncertainty in imaging outcomes and no difference in patient satisfaction between groups (Downs and Black = 89%, low risk of bias)
		**Clinician:** Back beliefs Survey Knowledge and Attitudes Questionnaire	MD 2.85; 95% CI 1.85 to 3.85 MD 0.48; 95% CI 0.13 to 0.83	
		**Quality of care:** Proportion imaging (any)	23.5% IG v 23.9% CG OR 0.77: 95% CI 0.47 to 1.26	
		Received any opioid medication	50.5% IG v 62.8% CG OR 0.57; 95% CI 0.38 to 0.85	
		Received any non-opioid medication	72% IG v 69.1% CG OR 1.52; 95% CI 0.98 to 2.35	
		**Health service:** LOS	4.05 IG v 4.1 CG MD − 0.28; 95% CI − 0.84 to 0.28	
		Admission hospital	15.9% IG v 15.4% CG OR 0.96; 95% CI 0.54 to 1.71	
		Admission SSU	12.4% IG v 11.8% CG OR 1.99; 95% CI 0.91 to 4.37	
Gumidyala 2021 [40] USA Observational before-after study	IG (*n* = 645): after the Centers for Disease Control and Prevention (CDC) guidelines for opioid prescribing were published CG (*n* = 361): before the release of CDC guidelines	**Quality of care:** Number of prescriptions (NSAIDs)	44% IG v 37% CG OR 1.31; 95% CI 1.01 to 1.70	There was a 37% reduction in the odds of receiving an opioid medication after the release of the CDC prescribing guidelines (Downs and Black = 56%, moderate risk of bias)
		Number of prescriptions (opioids)	34% IG v 45% CG OR 0.63; 95% CI 0.48 to 0.82	
Haig 2019 [41] USA Observational before-after study	IG (*n* = 200): immediately after implementation of multifaceted LBP protocol which included clinician education, a new patient intake form for back pain management, an ED champion, regular clinician emails modeling best practices and optimal patient communication and early outpatient review CG (*n* = 200): before implementation of the protocol	**Quality of care:** Proportion of imaging	IG 51% v CG 49% OR 1.05; 95% CI 0.67 to 1.66	A complex consultation process to implement a LBP protocol that was mostly clinician-targeted demonstrated uncertain effects on imaging and opioid use with no difference in ED LOS (Downs and Black = 73%, low risk of bias)
		Proportion of opioid use	IG 33% v CG 39% OR 0.82; 95% CI 0.49 to 1.36	
		**Health service:**LOS	IG 3.84 v CG 3.82 *p* = 0.81	
Tracey 1994 [22] Ireland Observational before-after study	IG (*n* = 128): after the release of locally developed imaging guidelines which included change in policy so that imaging technicians were not to accept requests where appropriate reasons were not provided CG (*n* = 184): 2 months before guidelines were released	**Quality of care:** Proportion of imaging	IG 27.2% v CG 48.4% *p* = 0.0002	Locally developed imaging guidelines significantly reduced referral rates for lumbar spine radiography (implemented system change to support use of guidelines that limited access to radiography). High baseline imaging rates were reported (Downs and Black = 48%, high risk of bias)
*Studies evaluating health service-targeted interventions*				
Baker 1987 [52] USA Observational before-after using time series analysis	IG (*n* = 759): implementation of policy that saw a change in requirements for lumbar spine imaging requests (alteration to imaging request form) CG (*n* = 1443): before restrictions were applied to imaging requests	**Quality of care:** Number of radiographs (total)	IG 759 v CG 1443 (47% absolute reduction)	There was a 47% absolute reduction in total radiographs between IG and CG with an increase in clinically significant positive findings on the imaging that was done (Downs and Black = 36%, high risk of bias)
Buller-Close 2003 [57] USA Controlled interrupted time series study	IG (n = 258): phase 2 “on” alternative charting system with computerized CDS embedded in the electronic medical record (EDECS) CG1 (*n* = 103): phase 1 “off” (usual care before implementation of charting system) CG2 (*n* = 125): phase 3 “off” (charting system removed)	**Quality of care:** Quality of documentation (% essential items contained in medical record)	IG 90% v CG1 58% Absolute difference 32%, 95% CI 26 to 38	Appropriateness of documentation, testing, and treatment improved substantially with the implementation of the EDECS but not sustained when removed (Downs and Black = 32%, high risk of bias)
		Quality of documentation (% of essential items contained in discharge instructions)	IG 92% v CG1 57% Absolute difference 35%, 95% CI 31 to 39	
		Appropriateness of testing	IG 73% v CG1 59% Absolute difference 14%, 95% CI 1 to 27	
		Appropriateness of treatment	IG 85% v CG1 48% Absolute difference 37%, 95% CI 18 to 56	
Davies 2022 [45] Malta Retrospective observational cohort study	IG (*n* = 164): implemented an alternative care pathway with a physiotherapist in ED to treat LBP CG (*n* = 961): participants seen by a medical clinician (usual care)	**Quality of care:** Proportion X-ray	IG 12.8% v CG 14.2% OR 0.89, 95% CI 0.54 to 1.46	Physiotherapy management of LBP in ED had similar outcomes to medical management (uncertain effect on the proportion of imaging) (Downs and Black = 46%, high risk of bias)
Day 1995 [54] USA Non-randomized controlled study (interrupted time series design)	IG (*n* = 259): alternative charting system with computerized clinical guidelines embedded in the electronic medical record (EDECS) CG (*n* = 103): phase 1 (usual care before implementation of charting system)	**Quality of care:** X-ray ordered	IG 58% v CG 61% OR 0.87, 95% CI 0.55 to 1.39	Uncertainty whether computerized charting system reduced X-ray orders or narcotic use (Downs and Black = 36%, high risk of bias)
		Narcotic given	IG 30% v CG 41% OR 0.63, 95% CI 0.39 to 1.01	
De Gruchy 2015 [46] Australia Prospective observational cohort study	IG (*n* = 120): implemented an advanced practice physiotherapist in ED to treat LBP CG (*n* = 700): seen by a medical clinician (usual care)	**Health service:** Treatment time (LOS/hours)	IG 1.58 v CG 3.55 MD − 2.05, 95% CI − 2.19 to − 1.91	Participants treated by Advanced Practice Physiotherapists in ED had significantly shorter LOS and were more likely to be discharged home (Downs and Black = 64%, moderate risk of bias)
		Discharged home	IG 87.5% v CG 59.4% Adjusted OR 6.2, 95% CI 3.6 to 10.7	
Gallagher 1998 [44] USA Observational before-after using time series analysis	IG (*n* = 442): 10 years after the implementation of policy that saw a change in requirements for lumbar spine imaging requests CG (n = 759): before the implementation of new imaging request requirements	**Quality of care:** Proportionate difference in imaging (imaging: all ED presentations)	IG 442 X-rays (0.8%) v CG 759 X-rays (1.1%) Adjusted for ED volume—28% proportionate decrease in imaging between IG and CG (95% CI 20–36%)	There was a 28% proportionate decrease in lumbosacral X-rays 10 years after altering the imaging order forms (Downs and Black = 64%, moderate risk of bias)
Kim 2021 [47] USA Prospective observational cohort study	IG (*n* = 43): ED physical therapy (usual care + ED physical therapy consisting of: bedside assessment, education, goal-setting, active strategies to reduce pain, diagnosis-specific home exercises that maximize early mobilization, treatment-based classification system, customized home exercise program consisting of 3 exercises, follow-up appointment arranged 1 week after ED visit) CG (*n* = 58): usual care (any ED testing or treatment not involving ED physical therapist per the treating physician's usual and customary practice)	**Quality of care:** Opioids prescribed at ED visit	IG 20.9% v CG 27.6% OR 0.69, 95% CI 0.27 to 1.77	Uncertainty whether alternative physical therapy pathway reduces imaging or opioid use. No difference in ED LOS. (Downs and Black = 57%, moderate risk of bias)
		Non-opioids prescribed at ED visit	IG 83.7% v CG 63.8% OR 2.92, 95% CI 1.11 to 7.71	
		Number of imaging	IG 32.6% v CG 43% OR 0.64, 95% CI 0.28 to 1.45	
		**Health service:** ED LOS (hours)	IG 3.84 v CG 3.82	
Lau 2008 [55] China RCT	Intervention (*n* = 55): provision of “early physiotherapy” in the ED (provision of physiotherapy within 24 h of the onset of LBP including pain management, education, resources and interferential therapy) CGn (*n* = 55): standard medical ED care + walking training/aids as needed to be discharged	**Patient:** Pain (Mean NPRS at discharge from ED)	IG 5.6 v CG 7.2 MD -1.6, 95% CI -2.3 to 0.8	Slightly improved pain outcomes in the early physiotherapy group at discharge however these did not reach the level of clinical importance (2 points on NPRS). (Downs and Black = 93%, low risk of bias)
Miller 2015 [48] USA Retrospective observational cohort study	IG (*n* = 35): participants received an epidural steroid injection instead of being admitted to the hospital for medical pain control CG (*n* = 28): matched group (age, gender, pain severity) admitted to the hospital for medical pain control	**Quality of care:** Morphine equivalents (ED)	IG 322 v CG 608.50	
		**Health service:** ED LOS (hours)	IG 8 (SD3.6) v CG 13 (SD4.2) *p* ≤ 0.002	An epidural injection pathway of care improved total ED LOS and total dosages of pain medication required while in ED and at discharge. Significantly lower cost of care compared to those who were admitted for pain management (Downs and Black = 50%, moderate risk of bias)
		**Cost:**	IG $4800 (SD 2000) v CG $33,000 (SD 14000) *p* ≤ 0.001	
Sayer 2018 [49] Australia Retrospective observational cohort study	IG (*n* = 360): participants seen by an Advanced Musculoskeletal Physiotherapist in ED CG (*n* = 729): group seen by another health professional in ED (medical or nurse practitioner)	**Health service:** ED LOS (hours)	IG 2.4 v CG 2.9 *p* ≤ 0.001	Improved ED metrics were demonstrated in participants with LBP seen by Advanced Musculoskeletal Physiotherapists compared to those seen by medical and nurse practitioner clinicians (Downs and Black = 72%, low risk of bias)
		Hospital admission	IG 10% v CG 35% *p* ≤ 0.001	
		Achievement of NEAT	IG 93% v CG 76% *p* ≤ 0.005	
Schulz 2016 [50] Australia Prospective observational cohort study	IG (*n* = 19): implemented an Advanced Musculoskeletal Physiotherapist to treat LBP CG (*n* = 10): usual care (seen by other healthcare workers)	**Patient:** NPRS	IG 7.9 (SD 2.5) v CG 6.3 (SD 2.8) *p* = 0.148 IG 0% v CG 30% *p* = 0.042	Advanced Musculoskeletal Physiotherapists ordered less imaging than other health professionals (Downs and Black = 68%, moderate risk of bias)
		**Quality of care:** Imaging NSAIDs	IG 58% v CG 80% *p* = 0.234	
Venkatesh 2021 [20] USA Prospective observational study	IG (*n* = 104 EDs): EDs participating in the Emergency Quality Network (E-Qual) avoidable imaging initiative in 2018 CG (*n* = 104 EDs): EDs participating in the E-QUAL initiative in 2017	**Quality of care:** X-ray rates	IG 33.3% v CG 36–2.7% (95%CI; − 5.9 to − 0.5%) *p* = 0.095	There was no significant change in XR, CT, or MRI utilization for LBP for EDs that participated in the quality improvement initiative (Downs and Black = 48%, high risk of bias)
*Studies evaluating interventions that target more than one health system level*				
Angus 2020 UK Observational before-after study	IG (*n* = 1477): 3 years after implementation of an alternative model of care “atraumatic back pain pathway” led by a consultant physiotherapist including a new LBP assessment proforma with CDS, clinician education and stakeholder mentorship CG (*n* = NR): 3 years before implementation of pathway “usual care”	**Health service:** Emergency short stay admissions	IG 556.3/year v CG 821.5/year	Authors report fewer admissions to short stay and a reduction in LOS of 25% after implementing a consultant-physiotherapy-led atraumatic LBP pathway in ED (Downs and Black = 36%, high risk of bias)
		LOS	IG reduced by 25% v CG	
Meisel 2022 USA RCT	IG (*n* = 433): personalized opioid risk communicated via opioid risk tool visual aid and video narratives CG (*n* = 434): generalized opioid risk information (information sheet including benefits, side effects, and risks of various analgesic options including opioids)	**Patient:** NPRS (during ED)	IG 6.5 (SD 2.2) v CG 6.8 (SD 2.2)	Probabilistic risk tool + narrative was more effective than general risk information in decreasing preference for opioids at discharge, satisfaction with pain treatment, and shared decision-making. Uncertainty whether the risk tool + narrative interventions reduced odds of receiving an opioid medication in ED. Opioid risk tool patients spent 25 min less time in the ED than patients who received general risk information (Downs and Black = 75%, low risk of bias)
		Analgesia preference (opioids) Satisfaction with	IG 25.9% v CG 33% Difference − 7.00, 95% CI − 13.1 to − 1.0	
		pain treatment (Mean—1 day post)	IG 7.25 v CG 6.57 Difference 0.69, 95% CI 0.08 to 1.29	
		Alignment of patient preference with clinician prescription	IG 70.1% v CG 65.9% Difference 4.2, 95% CI − 2.0 to 10.4	
		**Quality of care:** Opioids given in ED (*n*/%)	IG 23.1% v CG 28.8% OR 0.74, 95% CI 0.55 to 1.01	
		**Health service:** LOS (hours)	IG 3.83 v CG 4.08 MD − 0.25, 95% CI − 0.36 to − 0.14	
Min 2017 Canada Observational before-after study	IG (*n* = NR): after implementation of a point-of-care checklist of accepted “red flags” for LBP embedded into computerized order entry form for lumbar imaging CG (*n* = NR): before implementation of an electronic CDS tool (usual care)	**Quality of care:** Proportion imaging (imaging: total LBP visits)	IG 17% v CG 22% *p* = 0.0002	After implementing a red flag checklist in the order entry form for lumbar imaging, the study observed a 22% relative decrease in the median rate of imaging of LBP patients in the ED (Downs and Black = 52%, moderate risk of bias)
Peters 2022 Belgium Observational before-after study	IG (*n* = NR): after implementation of a new LBP protocol based on evidence-based guidelines and in keeping with organizational aspects of the ED and hospital CG (*n* = NR): before implementation of the protocol (usual care)	**Quality of care:** Imaging rates	IG 14–14.6% CT use and 12.7–13.5% X-ray use v CG CT and X-ray use > 25% Absolute reduction of around 10%	Observed a significant decrease of both percentages from over 25% before implementation of the new protocol to 14–14.6% for CT scan use and 12.7–13.5% for X-ray use after the introduction of a compulsory eLearning package (Downs and Black = 42%, high risk of bias)
Sapadin 2022 USA Observational before-after study	IG (*n* = NR): 8 months after multifaceted quality improvement intervention to improve the appropriateness of lumbar imaging CG (*n* = NR): 3 months before quality improvement intervention being implemented (usual care)	**Quality of care:** % of appropriately ordered radiographs	IG 53.2% v CG 5.8% Absolute increase in appropriateness of 47.4%	A multi-component QI intervention led by a clinical champion resulted in a reduction in the number of radiographs ordered to evaluate LBP and an increase in the proportion of those ordered that were appropriate (total number decreased by almost 44% and of those that were ordered, the appropriate proportion increased by tenfold) (Downs and Black = 46%, high risk of bias)
		Absolute number of radiographs	IG 47/month v CG 90/month Absolute reduction in imaging of 52%	
Tacy 2017 USA Observational before-after study	IG (*n* = 89): participants seen in pilot period 3 months after implementation of protocol CG (*n* = 46): participants seen in prelaunch period immediately preceding the pilot (1 month before)	**Patient:** Pain reduction at discharge (> 20% reduction)	IG 45% v CG 37.4%	90% of patients were discharged with guideline-based prescriptions after the launch of the protocol. Further work is needed on discharge instructions with only 45% of patients discharged with guideline-based advice. Patients were satisfied (Downs and Black = 43%, high risk of bias)
		**Quality of care:** DC prescriptions in line with guidelines	IG 90% v CG 82.6%	
		Back exercise instructions are given at DC	IG 45% v CG 37%	
		Referral to specialty	IG 30% v CG 23.9%	

*IG* intervention group, *NR* not reported, *CG* comparison group, *OR* odds ratio, *HIE* health information exchange, *CDS* clinical decision support, *SSU* short stay unit, *NPRS* Numerical Pain Rating Scale, *SD* standard deviation, *NEAT* National Emergency Access Target, *NSAIDs* non-steroidal anti-inflammatories, *GP* general practitioner, *DC* discharge

### Participant characteristics

Participant and LBP characteristics were not consistently reported across all studies. Ten studies did not report participant age or sex [19–22, 37, 42, 45, 52, 54, 57]. The average age among the other 18 studies ranged from 29 to 52.7 years, with 50% female. Most of the studies did not specify whether a participant had acute, subacute, or chronic LBP. Sixteen studies included participants with LBP-related leg symptoms [14, 17, 19, 21, 37, 38, 41, 42, 44–46, 50–52, 55, 58], and eight studies included participants with concerning features for serious spinal conditions (e.g., progressive neurological compromise, malignancy, infection) [17, 21, 22, 37, 41, 42, 44, 52]. These LBP characteristics of participants were unclear in 13 studies [19, 20, 22, 39, 40, 43, 46–49, 54, 56, 57].

### Intervention characteristics—health system target and implementation strategies used

Most of the studies (*n* = 22) included interventions that were targeted within a single system of LBP care provision (patient, clinician, or health service). Two of these studies evaluated interventions targeted at patients with LBP presenting to ED [17, 58], eight studies evaluated clinician-targeted interventions [14, 22, 38–41, 51, 53] and 12 studies evaluated interventions that targeted health service delivery [20, 44–50, 52, 54, 55, 57]. Six studies implemented multiple-level LBP interventions using strategies that were focused across more than one level of health service provision [19, 21, 37, 42, 43, 56].

We identified 44 individual strategies that were used to support implementation of the interventions (see online resource; Table S3). Strategies that aimed to train and educate clinicians or patients on LBP best practice were the most frequently used (31% of strategies). Sixteen studies incorporated at least one systems-based strategy into their intervention. The most common systems-based changes included the implementation of a new clinical pathway, most of which incorporated Physiotherapists in ED to treat LBP [19, 37, 45–47, 49, 50, 55], alterations to imaging order processes based on guideline-informed appropriate indications [21, 22, 44, 52] and the use of computerized medical records with embedded clinical decision support [42, 54, 57].

### Risk of bias

**Fig. 2:**
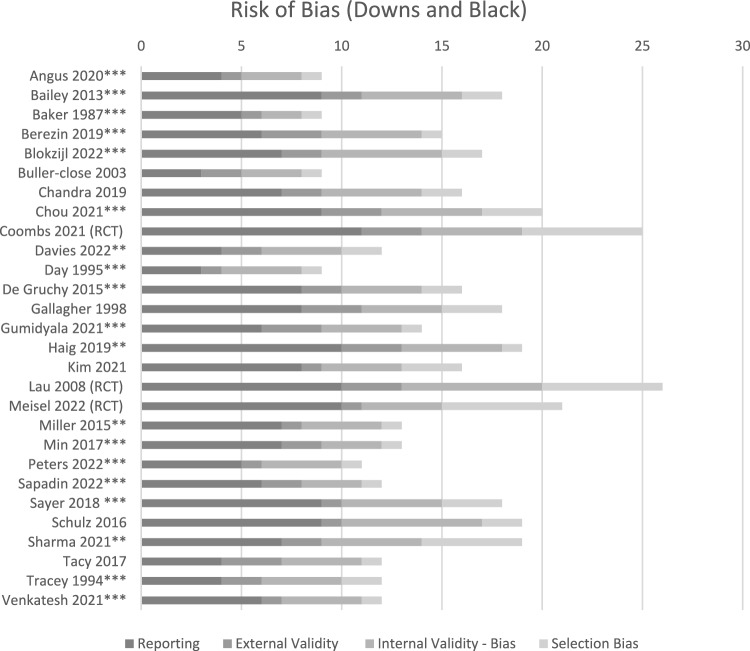
Risk of Bias assessment using Downs and Black checklist. ***Denominator for assessment reduced to 25 due to some items being not applicable to study design. **Denominator for assessment reduced to 26 due to some items being not applicable to study design. *RCT* randomized controlled trials

The Cochrane Risk of Bias assessments of the two RCTs and one cluster-randomized trial [14, 55, 56] are included in the online resource; Table S4. When comparing the three RCTs to the non-RCTs on the Downs and Black checklist, the RCTs scored between 75 and 93% and non-RCTs scored 32–80%. Overall, 10 out of the 28 included studies were assessed to be at high risk of bias (scores ≤ 50%) (Fig. 2). Studies scoring poorly were due to lack of randomization, poor identification and/or adjustment for potential confounding factors in the main analysis, not reporting adverse events, and not detailing recruitment processes and the total source population from which the study sample was derived. Eight studies scored above 70% on the Downs and Black checklist (i.e., low risk of bias scoring). Three of these were observational studies, achieving higher scores through comprehensive reporting and adjustments for confounding factors and reported adverse events.

### Effects of interventions

#### Primary outcome: quality of care (lumbar imaging)

**Fig. 3:**
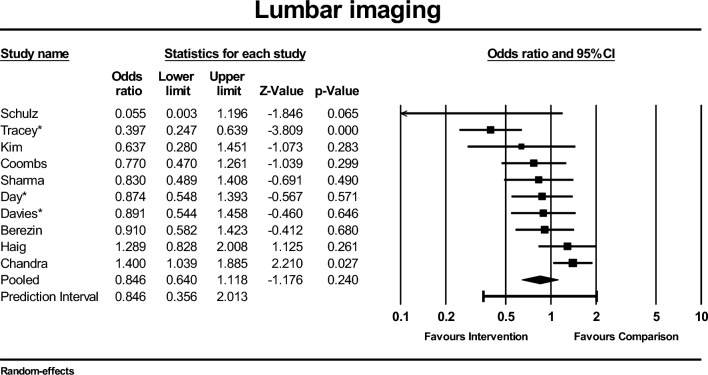
Forest plot of comparison. Outcome: lumbar imaging. *Studies assessed to be high risk of bias

Ten of the 19 studies that reported the outcome of lumbar imaging rates presented data that could be pooled in a meta-analysis (Fig. 3). One study was excluded as all participants had received lumbar imaging at a prior ED visit [51]. There is uncertainty whether interventions reduce lumbar imaging in ED (OR 0.85; 95% CI 0.64–1.12; *I*^2^ = 66%, 10 studies, 9804 participants, GRADE: very low-certainty evidence). The prediction interval for the distribution of true effects of interventions on lumbar imaging in 95% of adults with LBP in ED ranged between 0.36 and 2.01 (see online resource; Fig. S1).

There were no significant differences seen in effect sizes across the subgroups, except for baseline imaging rates and systems-based changes (see online resource; Table S5). For the three studies that reported high baseline imaging rates (i.e., ≥ 36%) [22, 47, 54], the intervention significantly reduced imaging rates (OR 0.60; 95% CI 0.39–0.93), whereas the effect was uncertain in the seven studies that reported low baseline imaging rates (i.e., < 36%) (OR 0.99; 95% CI 0.76–1.31) [14, 17, 38, 39, 41, 45, 50]. The *Q* value of the between subgroup comparison was 3.71 with a *p* value of 0.05 (< 0.10) demonstrating a significant difference in effect size between subgroups for baseline imaging rates. For the five studies that included systems-based changes in their intervention [22, 45, 47, 50, 54], the intervention reduced imaging rates (OR 0.65; 95% CI 0.45–0.94), whereas the effect was uncertain in the five studies that did not incorporate systems-based changes (OR 1.04; 95% CI 0.77–1.41) [14, 17, 38, 39, 41]. The *Q* value of the between subgroup comparison was 3.85 with a *p* value of 0.05 (< 0.10) demonstrating a significant difference in effect size between subgroups for systems-based changes.

### Primary outcome: quality of care (opioids and non-opioids)

**Fig. 4:**
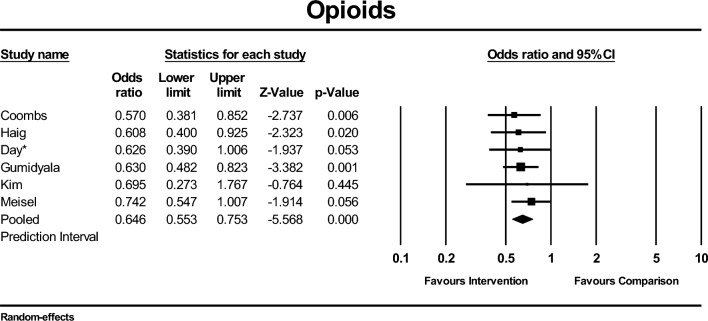
Forest plot of comparison. Outcome: opioids. *Studies assessed to be high risk of bias

Six of the eight studies that reported the number of participants who were given an opioid medication in ED reported results that could be pooled in a meta-analysis. Compared to the comparison group, there was a reduction in the odds of receiving an opioid medication in the group exposed to the intervention (OR 0.65; 95% CI 0.55–0.75; *I*^2^ = 0%, six studies, 7361 participants, GRADE: low-certainty evidence) (Fig. 4).

The prediction interval was not calculated, and subgroup analyses were not undertaken for opioid use because the between-study variance is estimated to be zero with less than ten studies included. There were only three studies that reported the number of participants who received a non-opioid medication in ED [14, 40, 50] (see online resource; Fig. S2). There is uncertainty whether interventions increase non-opioid use in ED (OR 1.33; 95% CI 0.99–1.79; *I*^2^ = 22%, three studies, 5660 participants, GRADE: very low-certainty evidence). Subgroup analyses were not conducted due to the small number of studies included in this analysis.

### Secondary outcome: health service (ED LOS)

Four of seven studies that reported the outcome of ED LOS presented data that could be pooled in meta-analysis (see online resource; Fig. S3) [14, 47, 49, 56]. One study was excluded from the LOS analysis as they only included participants who were being considered for admission due to their symptoms not responding to ED pain management, which included IV opioids [48]. Compared to participants not exposed to the intervention, there was a small reduction in ED LOS in participants exposed to the LBP interventions (MD − 0.38 h; 95% CI − 0.58 to − 0.17; *I*^2^ = 33%, 4 studies, 6682 participants, GRADE: low-certainty evidence). The prediction interval for the distribution of true effects of interventions on ED LOS in 95% of adults with LBP ranged between − 1.07 and 0.32 (see online resource; Fig. S4).

### Secondary outcome: health service (hospital admission)

Only three studies reported hospital admission outcomes (one cluster-randomized trial, two observational studies, 6534 participants) [14, 46, 49], with variation in how this outcome was defined (hospital admission and/or emergency short stay admissions). Therefore, this outcome is presented narratively. A large cluster-randomized trial evaluated a multifaceted clinician-targeted intervention to implement a LBP model of care in ED [14]. The study demonstrated uncertain effects on either hospital (OR 0.96; 95% CI 0.54–1.71) or emergency short stay unit admissions (OR 1.99; 95% CI 0.91–4.37). The two observational studies both evaluated an alternative pathway of care for LBP led by advanced practice musculoskeletal physiotherapists (AMPs) [46, 49]. Both studies reported large reductions on the odds of hospital admission in the group treated by AMPs (OR 0.21; 95% CI 0.12–0.36 [46] and OR 0.20; 95% CI 0.14–0.30 [49]) respectively. Overall, the certainty of evidence on hospital admission was very low.

### Secondary outcome: patient-reported

Nine studies evaluated patient-level outcomes [14, 17, 37, 43, 47, 50, 55–57], with patient satisfaction reported in eight of these studies. However, the methods used to measure patient satisfaction were variable with pooling of results not possible. Six studies reported positive effects on patient satisfaction in the group exposed to the LBP intervention [37, 43, 50, 55–57] with the remaining two studies reporting no difference in satisfaction between groups [14, 17]. There was significant heterogeneity in other outcome measures used to evaluate the effect of interventions on patient-level outcomes across the studies.

## Discussion

Our review identified 28 studies that aimed to improve LBP care in ED and pragmatically grouped these based on the health system level their interventions targeted. Interventions were mostly single-system focused with a preference for education-based implementation strategies targeting patients or clinicians. Our findings suggest that interventions can reduce the odds of a person with LBP receiving an opioid medication while in ED, but there is uncertainty around the effects of interventions on ED imaging practices. However, there was a greater effect on imaging in studies that have a high baseline imaging rate (> 36%) and interventions that include systems-based strategies. In addition, although not as consistently reported, ED LBP interventions do not appear to have negative effects on health service outcomes, including LOS, or patient outcomes such as satisfaction with care. Overall, effect estimates should be interpreted with caution as the certainty of evidence across all outcomes was deemed low to very low due to a lack of high-quality RCTs evaluating LBP implementation interventions in the ED setting.

Two previous reviews also reported limited evidence of the effectiveness of imaging interventions for LBP [59, 60], however, did not report other ED-relevant outcomes. Similar to our findings, the interventions aiming to reduce imaging rates were mostly single-system targeted and were mostly aimed at changing clinician behavior through education-based strategies. Multiple factors at patient, clinician, and health service levels influence ED LBP care decisions [61]. As such, interventions that target clinician behavior alone may be less likely to improve overall ED compliance with recommended guidelines. We could only identify six studies [19, 21, 37, 42, 43, 56] that used strategies targeting more than one health system level. While four of these studies demonstrated promising results in improving imaging outcomes, they were unable to be used in meta-analysis due to insufficient reporting of participant numbers in each group [19, 21, 37, 42]. The potential of interventions targeted at multiple levels of the health system was also reported in a review of Choosing Wisely interventions on low-value medical services such as imaging (33), with interventions targeting more than one level more commonly resulting in positive changes to imaging practices.

Lumbar imaging was the most common quality of care outcome across our included studies. However, only half of the studies that reported lumbar imaging outcomes reported data that could be used in the meta-analysis, including only one cluster RCT [14]. The prediction interval for true effects on lumbar imaging demonstrates substantial variation in the effect size across comparable LBP populations. We performed subgroup analyses to explore these variations and observed that baseline imaging rates and systems-based strategies may be important to consider in future studies that target lumbar imaging in ED.

There was a larger effect in studies that reported higher baseline imaging rates (> 36%) (OR 0.60; 95% CI 0.39–0.93) compared to studies with low baseline imaging rates (OR 0.99; 95% CI 0.76–1.31). In ED populations with already low baseline lumbar imaging rates, there may be little room for improvement, although we do not yet understand what the optimal imaging rate for people with LBP in ED is. With a higher prevalence of serious pathology than in primary care settings [16], a certain number of imaging investigations will be appropriate. As such, imaging appropriateness measured against LBP guidelines would be a more suitable reflection of ED performance than imaging rates alone. Only two included studies evaluated imaging appropriateness, both reporting favorable results however the methods used to define imaging appropriateness were inconsistent and had not been previously validated [21, 57]. Methodology to evaluate imaging appropriateness (overuse and underuse) in ED has been published [15, 62] and should be considered in future ED LBP research.

We also observed a more positive effect on lumbar imaging rates in the five studies that incorporated systems-based changes. These studies used strategies such as new clinical care pathways involving physiotherapists in ED [45, 47, 50], an alternative charting system with computerized clinical guidelines [54], and changes to an imaging policy [22]. Other studies that included systems-based changes to their intervention to improve lumbar imaging were identified in our review and reported promising results but were unable to be included in the meta-analysis due to insufficient reporting of the total source population [44, 52]. By altering an imaging request form to limit the acceptable indications for lumbar imaging, Baker et al. [52] reported an absolute reduction in imaging requests of 47%, with Gallagher et al. [44] reporting the sustainability of this reduction 10 years after implementing the system change. Strategies such as altering imaging requesting processes from a health service level may better support the ED clinician to change their imaging behavior and facilitate improved lumbar imaging rates. However, more high-quality studies are needed to further explore their effects.

Although imaging was not changed, opioid use was reduced in groups exposed to ED LBP interventions. These varying effects of interventions to improve different aspects of LBP care may partly be explained by how barriers to imaging and opioids were addressed in the different strategies. Clinician conviction to “do something” for their patient with LBP has been identified in multiple studies as a barrier to providing guideline-based care [19, 23, 66]. Recommending that certain treatments are not used, without providing ED clinicians an alternative option, is unlikely to support them to change care practices. Opioid-alternative pain management strategies, such as simple analgesics, heat wraps, or the use of an opioid risk tool, are tangible options and may have accounted for the positive effect on opioid outcomes [14, 56]. Providing an alternative to imaging, however, is likely more difficult. With pressures that encourage excessive imaging also coming from clinicians' fear of missing serious pathology [14, 20, 61, 66], safety net strategies such as early outpatient follow-up clinics, may be another promising strategy to explore. Providing ED clinicians with the option of referring or diverting suitable patients with LBP to timely outpatient follow-up clinics [63], where further screening for serious or specific pathology can occur, may provide the reassurance needed for safe discharge from ED without imaging.

### Strengths and limitations

This is the first systematic review to investigate LBP implementation interventions at different health system levels and their effects on multiple outcomes in the ED setting. Evaluating the effects of individual interventions was not the aim of this review. Rather, we have categorized interventions into patient, clinician, health service, or multiple levels. The ACSQHC LBP clinical care standards [5] describe key components of appropriate care across patient, clinician, and health service levels. Additionally, factors influencing LBP care in the ED have also been identified within each of these system levels [61]. The use of implementation frameworks and formative evaluation to identify and address local system barriers and enablers have also been outlined as key considerations to improving the quality of LBP implementation research design [64, 65]. As such, we have reviewed interventions through a health system lens to explore strategies within these different levels of ED LBP care provision. This broad approach will guide the systematic design of future LBP implementation research in the ED setting. However, a limitation of this approach, is that we combined data from different study designs testing different interventions if they were evaluating the same outcome. This may have contributed to the heterogeneity observed between the studies. There was also clinical heterogeneity across our included studies with participants having different LBP characteristics, with many studies not reporting in what proportion (e.g., some studies included participants with LBP-related leg symptoms and/or serious spinal conditions and some specifically excluded these participants). These variations may influence decisions around imaging and the use of pain medicines in ED. However, it was important for us to include participants with these different LBP features because the 2022 Australian Low Back Pain Clinical Care Standard [5] outlines recommended management for patients with acute LBP, with or without LBP-related leg symptoms, and includes patients with signs of serious pathology. We addressed this heterogeneity using subgroup analyses and prediction intervals to explore the variation in effect size across different study, participant, and intervention characteristics. There were no significant differences seen in effect sizes across all subgroups, except for baseline imaging rates and systems-based changes within the imaging outcome.

We built on a previous review of five studies evaluating ED imaging interventions [60], identifying an additional 23 studies. Therefore, we are confident that our rigorous, systematic search strategy included all relevant articles to date. However, non-English articles were excluded, and we did not measure publication bias, although varied findings suggest this to be minimal. Due to inadequate reporting of the total number of presenting patients, and no response from corresponding authors, many studies were unable to be included in the meta-analyses. Some of these studies reported promising interventions, which may have changed the effect estimate if they were able to be included. The limited number of studies reporting suitable data also required combining the small number of RCTs with non-RCTs in meta-analysis. Further, using the GRADEpro software we downgraded the certainty of evidence across all outcomes to either low or very low due to many studies being observational in design (see online resource; Table S6). However, this may underestimate the risk of bias as we assessed eight studies as low risk, including three large RCTs.

## Conclusion

We have demonstrated that interventions can improve LBP care in ED, primarily by reducing the use of opioids. However, the certainty of evidence is low, and more high-quality studies are required which should include consistent outcomes that are relevant to the ED setting. Future interventions and outcomes should not just focus on imaging and opioid use but encompass other key domains of recommended LBP care, such as psychosocial screening and appropriate referral pathways. Consideration of appropriateness outcomes that better reflect ED performance against LBP guidelines is also recommended. Most interventions reported in our review target one system of care, with few studies applying theory to identify and link barriers and enablers to intervention strategies. There is a need for well-designed interventions that are tailored to overcome ED-specific barriers to adopting guideline-based care and take a systems approach to better address the ongoing problem of improving LBP care in this setting.

## Supplementary Information

Below is the link to the electronic supplementary material.

Supplementary file1 (DOCX 175 KB)

## Data Availability

Data will be available upon request to the corresponding author.
