# Supplementary material for: Interventions to improve the quality of low back pain care in emergency departments: a systematic review and meta-analysis
Source: Intern Emerg Med. 2024 Sep 9;19(7):2057–76. doi: 10.1007/s11739-024-03736-y (PMC11466992; doi:10.1007/s11739-024-03736-y)
Supplement: Supplementary file 1 — Supplementary file1 (DOCX 175 KB) [file 11739_2024_3736_MOESM1_ESM.docx]

**TABLE S1:** PubMed search strategy

| **Search number** | **Query** | **Search Details** | **Results** | **Date** |
| --- | --- | --- | --- | --- |
| 1 | ((Emergency Service, Hospital[MeSH Terms]) OR (acute care[Title/Abstract])) OR (urgent care[Title/Abstract]) | "emergency service, hospital"[MeSH Terms] OR "acute care"[Title/Abstract] OR "urgent care"[Title/Abstract] | 120,728 | 12/01/2023 |
| 2 | ((Low Back Pain[MeSH Terms]) OR (spinal pain[Title/Abstract])) OR (lumbar pain[Title/Abstract]) | "low back pain"[MeSH Terms] OR "spinal pain"[Title/Abstract] OR "lumbar pain"[Title/Abstract] | 28,096 | 12/01/2023 |
| 3 | #1 AND #2 | ("emergency service, hospital"[MeSH Terms] OR "acute care"[Title/Abstract] OR "urgent care"[Title/Abstract]) AND ("low back pain"[MeSH Terms] OR "spinal pain"[Title/Abstract] OR "lumbar pain"[Title/Abstract]) | 192 | 12/01/2023 |

**TABLE S2:** Characteristics of the included studies (n = 28)

| **Studies evaluating patient-targeted interventions** | | | | | | |
| --- | --- | --- | --- | --- | --- | --- |
| **Study location and design** | **Participant LBP characteristics** | **Intervention and comparison** | **Implementation strategies** | **Outcome measures** | **Results** | **Study findings** |
| Chou 2021 USA Non-randomised controlled study | Adult (19-63 years) with discharge diagnosis related to LBP  *did not specify if leg pain was present   Excluded: Serious spinal or non-MSK pathology and re-presentations within 30 days | IG (n = NR): employer-mandated switch from low to high deductible health plan    CG (n = not reported): maintained low deductible health plan | **Patient-focused:**  *Financial strategies* - patient financial decentivising by increasing out of pocket cost for imaging | **Quality of care:**  Probability of low-value imaging during ED visit (low-value imaging / ED visits) | **Quality of care:**  Relative (%): -5.2, 95% CI -16.6 to 6.1 p = 0.37 | Good quality (DB 80%) Disincentivising patients through increasing out of pocket costs for imaging had limited effect on reducing low-value imaging during ED visits |
| Sharma 2021  Australia  Non-randomised controlled study (interrupted time series design) | Adults (≥18 years) presenting with "pain, back" triage code  *did not specify if leg pain was present or serious spinal conditions  Excluded:  Admitted patients, triage category 1 and 2, and those without mobile phones | IG (n = 99): potential harms of imaging displayed on LCD screens in ED waiting room plus provision of leaflet   CG (n = 238): usual waiting room LCD display plus leaflet accessible to patient | **Patient-focused:**  *Train and educate* - Five digital posters in different languages; patient leaflet on overdiagnosis and potential harms of imaging; used behavioural cues to discourage patients from having an imaging test (framing, cues to action and status quo)   **Clinician-focused:**  *Engage consumers* - Director of Emergency Medicine notified all clinical staff of the study via email | **Quality of care:**  Proportion of LBP presentations with at least 1 imaging test | **Quality of care:** 25% IG v 29% CG  OR 0.83, 95% CI = 0.49 to 1.41 | Good quality (DB 73%) Uncertain whether waiting room communication strategy can reduce lumbar imaging rates through increasing patient awareness of harms from unnecessary imaging |
| **Studies evaluating clinician-targeted interventions** | | | | | | |
| **Study and design** | **Participant LBP characteristics** | **Intervention and comparison** | **Implementation strategies** | **Outcome measures** | **Results** | **Intended, mixed or null/unintented results** |
| Bailey 2013  USA Retrospective observational cohort study | Adults (≥18 years) with a previous indexed ED visit for LBP with imaging with a discharge diagnosis of lumbosacral or thoracic back pain   Excluded:  Serious spinal or non-MSK pathology | IG (n = 100): electronic HIE accessed by ED staff during subsequent visit   CG (n = 700): did not have their electronic HIE accessed in ED | **Clinician-focused:**  *Support clinician* - health information exchange | **Quality of care:**  *Repeated* lumbar or thoracic diagnostic imaging (radiograph, CT, or MRI)   **Cost:**  Total patient-visit estimated cost | **Quality of care:** Repeated imaging 10% IG v 24.1% CG OR 0.36, 95% CI 0.18 to 0.71  **Cost:** $189 IG v $189 CG | Good quality (DB 72%) HIE use by both health care providers and ED support/nursing staff protected patients from repeated lumbar imaging (64% lower odds of any repeated imaging if HIE was accessed, even after controlling for other factors) |
| Berezin 2020  Canada Observational before-after study | Adults (18-70 years) with discharge diagnosis code of non-traumatic LBP including radicular back pain   Excluded:  Patients admitted to hospital, trauma, serious spinal pathology | IG (n = 515): after national release of Choosing Wisely Canada imaging guidelines   CG (n = 545): before release of Choosing Wisely Canada imaging guidelines | No active implementation strategies (passive dissemination of guidelines only, evidence-based guidelines target clinicians) | **Quality of care:**  Proportion of spinal imaging    Pathological findings found on imaging | **Quality of care:** 7.6% IG v 8.3% CG  OR 0.91, 95% CI 0.58 to 1.42   28.2% IG v 8.9% CG OR 4.03 95% CI 1.16 to 13.93 | Fair quality (DB 60%) - Guidelines did not change clinical practice (no effect on imaging rates, baseline imaging rates were very low)  - higher percentage of participants that did receive imaging in IG had positive findings reported |
| Blokzijl 2022  Australia  Observational before-after study using time-series analysis | Adults (≥18 years) with discharge diagnosis of non-specific LBP   Excluded:  Serious spinal or non-MSK pathology, radicular pain | IG (n = NR): after statewide release of LBP model of care    CG (n = not reported): before release of LBP model of care (usual care) | No active implementation strategies (passive dissemination of guidelines only, evidence-based guidelines target clinicians) | **Quality of care:**  Proportion of spinal imaging | **Quality of care:** 33.5% IG v 30.4% CG p = 0.37 | Fair quality (DB 68%) Interrupted time series data did not detect any significant differences in the level or slope of the trend in imaging following release of statewide model of care |
| Chandra 2019 Observational before-after study | Adults (27-70 years) with presenting complaint of back pain  *did not specify whether this included radicular back pain or serious spinal conditions | IG (n = 672): after implementation of LBP knowledge-translation initiative   CG (n = 781): before implementation of LBP knowledge-translation initiative (usual care) | **Clinician-focused:**  *Train and educate* - didactic education seminar (face-to-face and electronic); guideline recommendations on department website and posters in ED | **Quality of care:** Frequency of imaging | **Quality of care:** 16.2% IG v 12% CG OR 1.4, 95% CI 1.04 to 1.89 | Fair quality (DB 57%) Knowledge-translation intervention was associated with a subsequent increase in the rate of imaging for LBP |
| Coombs 2021  Australia  Stepped-wedge, cluster RCT | Adults (≥18 years) with a discharge diagnosis code of non-specific LBP or radicular back pain   Excluded: serious spinal conditions and re-presentations within 48 hours | IG (n = 1392): multifaceted intervention to implement new LBP model of care in ED  CG (n = 3233): cluster sites in control conditions (usual care) prior to implementation of intervention | **Patient-focused:**  *Train and educate* - patient handouts   **Clinician-focused:** *Train and educate* - education seminars held on numerous occasions over 4 week intervention period in the ED and teaching rooms; education materials including hardcopy of the model of care, a website, and decision support tools; posters highlighting key messages about benefits and harms of lumbar imaging, opioid medicines, and inpatient admission were displayed throughout ED   *Support clinicians* - non-opioid pain management options (heat wraps); CDS tool for appropriate use of lumbar imaging and analgesic medicines were distributed to clinicians; access to patient handouts for ease of education  *Use evaluative and iterative strategies* - audit and feedback dashboard with department-level outcome feedback and monthly newsletters also highlighting these data  **Health service-focused:**  *Stakeholder interrelationships* - able to fast-track patient referrals to outpatients | **Patient:**  ED patient experiences with care survey-item 31  **Clinician:**  Back beliefs Q  Knowledge and Attitudes Q  **Quality of care:**  Proportion imaging (any)  Received any opioid medication  Received any non-opioid medication    **Health service:**  LOS  Admission hospital  Admission SSU  Re-presentation (48 hours) | **Patient:** MD 0.16, 95% CI -0.72 to 1.03  **Clinician:** MD 2.85, 95% CI 1.85 to 3.85  MD 0.48, 95% CI 0.13 to 0.83   **Quality of care:** 23.5% IG v 23.9% CG  OR 0.77, 95% CI 0.47 to 1.26  50.5% IG v 62.8% CG  OR 0.57, 95% CI 0.38 to 0.85  72% IG v 69.1% CG  OR 1.52, 95% CI 0.98 to 2.35  **Health service:** 4.05 IG v 4.1 CG  MD -0.28, 95% CI -0.84 to 0.28  15.9% IG v 15.4% CG  OR 0.96, 95% CI 0.54 to 1.71  12.4% IG v 11.8% CG OR 1.99, 95% CI 0.91 to 4.37  1.3% IG v 1.2% CG  OR 0.31, 95% CI 0.06 to 1.57 | Good quality (DB 89%) - improved clinicians beliefs and knowledge regarding LBP and its management  - reduced odds of lumbar imaging (OR 0.77) but evidence is uncertain  - Absolute reduction in use of opioid medication of 12.3% from 62.8% to 50.5% (OR 0.57) - No effect on other healthcare utilisation outcomes - Intervention did not improve patient satisfaction however this was also not adversely affected by intervention even though opioid use was reduced |
| Gumidyala 2021  USA Before-after observational study | Adults (≥18 years) discharge diagnosis "mechanical back pain"  *did not specify if leg pain was present or serious spinal conditions  Excluded:  Pregnancy, LBP from non-MSK causes, patients admitted to hospital | IG (n = 645): after national release of opioid guidelines   CG (n = 361): before release of guidelines | No active implementation strategies (passive dissemination of guidelines only, evidence-based guidelines target clinicians) | **Quality of care:**  Number of prescriptions (non-steroidal anti-inflammatories)  Number of prescriptions (opioids) | **Quality of care:** 44% IG v 37% CG  OR 1.31, 95% CI 1.01 to 1.70  34% IG v 45% CG  OR 0.63, 95% CI 0.48 to 0.82 | Fair quality (DB 56%)  There was a 37% reduction in odds of receiving an opioid medication after release of the national opioid prescribing guidelines |
| Haig 2019 USA Before-after observational study | Adults (18-80 years) with discharge diagnosis of LBP or radicular back pain, including concerns for serious pathology | IG (n = 200): after implementation of multifaceted organisational consultation for back pain management   CG (n = 200): before implementation | **Patient-focused:**  *Train and educate* - education by clinicians with pamphlet  **Clinician-focused:**  *Support clinicians* - new LBP intake form for clinician use including a check-off history, physical examination, and order sheet  *Engage consumer*s - ED champion appointed; principal investigator did face-to-face reminders on the floor; clinician reminders via email   *Use evaluative and iterative strategies* - regular emails to clinicians with description of the program, copy of the patient brochure and 4 "case of the week" stories (modelling best practices and optimal patient communication)  *Train and educate* - 20-minute education session to clinicians; physical examination video made available; ED nursing staff were provided with education; posters   **Health service-focused:**  *Stakeholder interrelationships* - review by physiatry and physiotherapy within 48 hours - new process agreed upon by all stakeholder departments | **Quality of care:**  Proportion of imaging  Proportion of opioid use    **Health service:**  Representation to ED (within 1 month)  LOS | **Quality of care:**  IG 51% v CG 49%  OR 1.05, 95% CI 0.67 to 1.66  IG 33% v CG 39%  OR 0.82, 95% CI 0.49 to 1.36    **Health service:**  IG 12% v CG 16%  p = 0.02  IG 3.84 v CG 3.82 p = 0.81 | Good quality (DB 73%) A complex consultation process to implement a LBP protocol in ED demonstrated ability to positively change LBP management by reducing the odds of opioid use and representation rates, but the effect is uncertain. Unintended result of insignificantly increasing imaging in the IG compared to CG |
| Tracey 1994 Ireland  Before-after observational study | Acute LBP  *did not specify if leg pain was present or serious spinal conditions | IG (n = 128): after release of locally developed imaging guidelines  CG (n = 184): before guidelines released | **Clinician-focused:**  *Train and educate* - education to junior Doctors on baseline retrospective analysis results of imaging rates  **Health service-focused:** *Systems-based* - radiographers were not to accept requests where appropriate reason was not provided | **Quality of care:**  Proportion of imaging | **Quality of care:** IG 27.2% v CG 48.4%  p = 0.0002 | Poor quality (DB 48%) Locally developed imaging guidelines can significantly reduce referral rates for lumbar spine radiography (implemented system change to support use of guidelines that limited access to radiography) |
| **Studies evaluating health service-targeted interventions** | | | | | | |
| **Study and design** | **Participant LBP characteristics** | **Intervention and comparison** | **Implementation strategies** | **Outcome measures** | **Results** | **Intended, mixed or null/unintented results** |
| Baker 1987  USA Observational before-after using time-series analysis | Adult patients (≥18 years) presenting with LBP who received lumbar spine radiography (including serious pathology, trauma, and neurological compromise) *did not specify if leg pain was present  Excluded:  Pregnancy | IG (n = 759): implementation of policy that saw a change in requirements for lumbar spine imaging requests (alteration to imaging request form)  CG (n = 1443): before restrictions were applied to imaging requests | **Health service-focused:**  *Systems-based* - change to order set or documentation requirements with new imaging request forms that listed limited, specific indications for filming the lumbar spine; imaging guidelines embedded in new request forms (policy that imaging requests were declined if they did not list appropriate indications) | **Quality of care:**  Number of radiographs (total)  Frequency of clinically significant findings on imaging  **Implementation:**  Fidelity (compliance with radiograph forms) | **Quality of care:** IG 759 v CG 1443 (47% absolute reduction)  IG 13.4% v CG 9.1%  OR 4.3, 95% CI 1.6 to 7.3  **Implementation:** 79% used forms in IG | Poor quality (DB 36%) There was a 47% absolute reduction in total radiographs between IG and CG with an increase in clinically significant positive findings on the imaging that was done |
| Buller-Close 2003 USA Non-randomised controlled study (interrupted time series design) | 16 years or older presenting with LBP  *did not specify if leg pain was present or serious spinal conditions  Excluded:  chronic pain, back surgery in the last 2 years, known systemic disease or renal colic | IG (n = 258): alternative charting system with computerised clinical guidelines embedded in electronic medical record   CG (n = 103): usual care before implementation of charting system | **Health service-focused:**  *Systems-based* – change to order set or documentation requirements from handwritten to computerised charting system with clinical decision support tool; rationales for suggested tests and treatments provided; personalised printing of medical record, prescriptions, and discharge instructions | **Quality of care:**  % essential items contained in medical record  % of essential items contained in discharge instructions    Appropriateness of testing  Appropriateness of treatment  **Cost:**  (median charge per patient visit) | **Quality of care:** IG 90% v CG 58% Absolute difference 32%, 95% CI 26 to 38  IG 92% v CG 57%  Absolute difference 35%, 95% CI 31 to 39  IG 73% v CG 59%  Absolute difference 14%, 95% CI 1 to 27  IG 85% v CG 48%  Absolute difference 37%, 95% CI 18 to 56  **Cost:**  IG $439 USD v CG $411 USD  Absolute difference $28 USD, 95% CI -19 to 63 | Poor quality (DB 32%)  Appropriateness of documentation, testing and treatment improved substantially with implementation of the charting system (note effect was not sustained when system was removed)  Median charges increased by $28 |
| Davies 2022  Malta Retrospective observational cohort study | Patient presenting with LBP with radicular leg pain  Excluded:  Severe trauma, neurological deficits (weakness/cauda equina), pathology, paediatrics, and admissions | IG (n = 164): implemented alternative care pathway with physiotherapist in ED to treat LBP  CG (n = 961): participants seen by medical clinician (usual care) | **Health service-focused:**  *Systems-based* - creation of a new clinical pathway | **Quality of care:**  Proportion Xray | **Quality of care:** IG 12.8% v CG 14.2%  OR 0.89, 95% CI 0.54 to 1.46 | Poor quality (DB 46%) Physiotherapy management of LBP in ED had similar outcomes to medical management (no effect on proportion of imaging) |
| Day 1995  USA Non-randomised controlled study (interrupted time series design) | 16 years or older presenting with LBP  *did not specify if leg pain was present or serious spinal conditions  Excluded:  chronic pain, back surgery in the last 2 years, known systemic disease or renal colic | IG (n = 259): alternative charting system with computerised clinical guidelines embedded in electronic medical record   CG (n = 103): usual care before implementation of charting system | **Clinician-focused:**  *Train and educate* - clinicians educated on use of charting system  **Health service-focused:**  *Systems-based* - change to order set or documentation requirements from handwritten to computerised charting system with clinical decision support tool; rationales for suggested tests and treatments provided; personalised printing of medical record, prescriptions, and discharge instructions | **Quality of care:**  Xray ordered  Narcotic given | **Quality of care:** IG 58% v CG 61%  OR 0.87, 95% CI 0.55 to 1.39  IG 30% v CG 41%  OR 0.63, 95% CI 0.39 to 1.01 | Poor quality (DB 36%) Reduction in odds of receiving imaging or narcotic medication with use of electronic charting system however uncertainty in effect size estimate |
| De Gruchy 2015  Australia Prospective observational cohort study | Patients discharged with LBP ("sciatica, lumbar sprain/strain, lumbago and LBP")  *did not specify if serious spinal conditions were included | IG (n = 120): implemented advanced practice physiotherapist in ED to treat LBP  CG (n = 700): seen by medical clinician (usual care) | **Health service-focused:**  *Systems-based* - creation of a new clinical pathway | **Health service:**  Treatment time (LOS/hours)    Discharged home | **Health service:** IG 1.58 v CG 3.55  MD -2.05, 95% CI -2.19 to -1.91  IG 87.5% v CG 59.4%  Adjusted OR 6.2, 95% CI 3.6 to 10.7 | Fair quality (DB 64%)  Participants treated by Advanced Practice Physiotherapist in ED had significantly shorter LOS and were more likely to be discharged home |
| Gallagher 1998 USA Observational before-after using time-series analysis | Adults (≥18 years) presenting with LBP who received lumbar spine radiography (including serious pathology, trauma, and neurological compromise) *did not specify if leg pain was present  Excluded:  Pregnancy | IG (n = 442): 10-years after implementation of policy that saw a change in requirements for lumbar spine imaging requests   CG (n = 759): before implementation of new imaging request requirements | **Health service-focused:**  *Systems-based* – change to order set or documentation requirements with new imaging request forms that listed limited, specific indications for filming the lumbar spine; imaging guidelines embedded in new request forms (imaging requests were declined if they did not list appropriate indications) | **Quality of care:**  Proportionate difference in imaging (imaging/ all ED presentations) | **Quality of care:** IG 442 X-rays (0.8%) v CG 759 X-rays (1.1%)  Adjusted for ED volume - 28% proportionate decrease in imaging between IG and CG (95% CI 20-36%) | Fair quality (DB 64%)  There was a 28% proportionate decrease in lumbosacral X-rays 10 years after altering the imaging order forms |
| Kim 2021 USA Prospective observational cohort study | Adults (≥18 years) who presented to ED with a complaint of acute LBP (< 2 weeks)  *did not specify if leg pain was present  Excluded:  History of chronic LBP, non-English speaking, non-musculoskeletal, serious spinal conditions, and patients with high likelihood of hospital admission | IG (n = 43): ED physical therapy (usual care + ED physical therapy)  Comparison (n = 58): usual care (any ED testing or treatment not involving ED physical therapist in accordance with the treating physicians usual and customary practice) | **Health service-focused:**  *Systems-based* - creation of a new clinical pathway for access to physical therapy consisting of bedside assessment, education, goal setting, active strategies to reduce pain, diagnosis-specific home exercises that maximise early mobilisation, treatment-based classification system, customised home exercise program consisting of 3 exercises, follow up arranged 1 week after ED visit | **Patient:**  ODI baseline during ED (Med)  PROMIS-PI baseline (during ED)   **Quality of care:**  Opioids prescribed at ED visit  Non-opioids prescribed at ED visit   Number of imaging  **Health service:**  ED LOS (hours) | **Patient:** IG 51.1 v CG 36.0  IG 67.6 v CG 62.7  **Quality of care:**  IG 20.9% v CG 27.6%  OR 0.69, 95% CI 0.27 to 1.77  IG 83.7% v CG 63.8%  OR 2.92, 95% CI 1.11 to 7.71  IG 32.6% v CG 43%  OR 0.64, 95% CI 0.28 to 1.45  **Health service:** IG 3.84 v CG 3.82 | Fair quality (DB 57%)  Found an association between ED physical therapy pathway for acute LBP and improvements in pain-related functioning and reductions in analgesic medication usage compared with usual care. Reduction in odds of imaging with uncertainty in result |
| Lau 2008  China RCT | Adults (≥18 years) presenting with LBP with or without leg pain that commenced within the preceding 24 hours before presentation to ED   Excluded: concerns for serious spinal conditions, previous LBP, pregnancy, previous hip, or back surgery | Intervention (n = 55): provision of "early PT" in the ED  Comparison (n = 55): standard medical ED care + walking training/aids as needed to be discharged | **Patient-focused:**  *Train and educate* - resources provided to patients in the IG   **Health service-focused:**  *Systems-based* - creation of a new clinical pathway for very early access to PT (within 24 hours of LBP onset) consisting of pain management, education, resources, and interferential therapy | **Patient:**  Pain (Mean NPRS at discharge from ED) | **Patient:**  IG 5.6 v CG 7.2  MD -1.6, 95% CI -2.3 to 0.8 | Excellent quality (DB 93%) Slightly improved pain outcomes in intervention group at discharge however these did not reach level of clinical importance |
| Miller 2015  USA Retrospective observational cohort study | Patients with diagnosis of LBP that were being considered for hospital admission because they did not respond to treatment with NSAIDs, muscle relaxants and IV narcotic medication (symptoms of radiculopathy were included)  Excluded:  Serious spinal conditions, cancer, fracture or infection, non-musculoskeletal causes | IG (n = 35): participants received an epidural steroid injection instead of being admitted for medical pain control  CG (n = 28): matched group (age, gender, pain severity) admitted to the hospital for medical pain control | **Health service-focused:** *Support clinicians* - creation of a new clinical pathway involving provision of epidural steroid injection to prevent hospital admission | **Quality of care:**  Morphine equivalents (ED)  **Health service:**  ED LOS (hours)  **Cost** | **Quality of care:** IG 322 v CG 608.50  **Health service:**  IG 8 (SD 3.6) v CG 13 (SD 4.2)  p = <0.002  **Cost** IG $4800 (SD 2000) v CG $33000 (SD 14000) p = < 0.001 | Fair quality (DB 50%)  An epidural injection pathway of care improved total ED LOS and total dosages of pain medication required while in ED and provided as discharge prescriptions  Significantly lowered cost of care versus those that were admitted for pain management |
| Sayer 2018  Retrospective observational cohort study | Adults (18-65 years) who presented to the ED with LBP during the audit period  *did not specify if radicular back pain or serious spinal conditions were included | IG (n = 360): participants seen by Advanced Musculoskeletal Physiotherapist in ED   CG (n = 729): group seen by another health professional in ED (medical or nurse practitioner) | **Health service-focused:**  *Systems-based* - creation of a new clinical pathway | **Health service:** ED LOS (hours)  Hospital admission  Achievement of NEAT | **Health service:** IG 2.4 v CG 2.9  p = < 0.001  IG 10% v CG 35%  p = < 0.001  IG 93% v CG 76%  p = < 0.005 | Good quality (DB 72%)  Improved ED metrics were demonstrated in participants with LBP seen by Advanced Musculoskeletal Physiotherapists compared to those seen by medical and nurse practitioner clinicians |
| Schulz 2016  Australia  Prospective observational cohort study | Adults (18-65 years) presenting with acute musculoskeletal LBP <3 months duration (with or without leg symptoms)   Excluded:  patients requiring inpatient review or non-English speaking, serious spinal conditions, non-musculoskeletal | IG (n = 19): implemented an Advanced Musculoskeletal Physiotherapist to treat LBP   CG (n = 10): usual care (seen by other healthcare workers) | **Health service-focused:**  *Systems-based* - creation of a new clinical pathway | **Patient:**  NPRS  **Quality of care:**  Imaging (no/yes)  NSAIDs | **Patient:**  IG 7.9 (SD 2.5) v CG 6.3 (SD 2.8)  p = 0.148  **Quality of care:** IG 0% v CG 30%  p = 0.042  IG 58% v CG 80% p = 0.234 | Fair quality (DB 68)  Advanced Musculoskeletal Physiotherapists ordered less imaging than other health professionals however very small sample size with missing opioid data |
| Venkatesh 2021 USA Prospective observational study | Adults (18-65 years) with LBP diagnostic codes  *did not specify if leg pain was present or serious spinal conditions  Excluded: trauma, ED sites that did not participate in the quality improvement initiative over the 2-year period | IG (n = 104 EDs): EDs participating in the Emergency Quality Network (E-Qual) avoidable imaging initiative in 2018   CG (n = 104 EDs): EDs participating in the E-QUAL initiative in 2017 | **Health service-focused:**  Note participants were EDs not individuals   Common initiatives adopted by all EDs: - departmental protocol or guideline (99%) - collaboration with radiology (92-99%)  - engagement with leadership (97-100%) | **Quality of care:**  Xray rates  CT scan rates  MRI rates | **Quality of care:** IG 33.3% v CG 36%  -2.7% (95%CI; -5.9 to -0.5%) p = 0.095  IG 17.7% v CG 20.1%  -2.4% (95%CI; -5.1 to -0.4%) p = 0.09  IG 0.7% v CG 0.8%  -0.1% (95%CI; -0.4 to -0.3%) p = 0.777 | Poor quality (DB 48%)  There was no significant change in XR, CT or MRI utilization for LBP between 2017 and 2018 for EDs that participated in the quality improvement initiative (only included sites that participated in the initiative across both years) |
| **Studies evaluating interventions that target more than one health system level** | | | | | | |
| **Study and design** | **Participant LBP characteristics** | **Intervention and comparison** | **Implementation strategies** | **Outcome measures** | **Results** | **Intended, mixed or null/unintented results** |
| Angus 2020  UK Before-after observational study | Admitted to ED with "back pain, neck pain, radicular pain, radiculopathy, neurological deficit, suspected CES, myelopathy, spinal infection and spinal fracture" *Age not specified | IG (n = 1477): after implementation of an alternative model of care "atraumatic back pain pathway" led by consultant physiotherapist   CG (n = NR): before implementation of pathway "usual care" | **Clinician-focused:**  *Support clinicians* - development of a LBP assessment proforma with clinical decision-making aids; dedicated phone line for clinicians to access consultant physiotherapy team  *Train and educate* - education sessions to improve acute assessment and management of LBP   *Stakeholder interrelationships* - clinical mentorship with spinal surgeons, access to spinal meetings for education and case discussion, mentorship and support from ED and acute medicine physicians    **Health service-focused:**  *Systems-based* - creation of a new clinical pathway | **Quality of care:**  Emergency short stay admissions  LOS  Return rates (30-day) | **Quality of care:**  IG 556.3/year v CG 821.5/year  IG reduced by 25% v CG  IG 0.8% v CG 1.2% | Poor quality (DB 36%) Report less admissions to short stay and reduction in LOS of 25% after implementing a consultant-physiotherapy led atraumatic LBP pathway in ED |
| Meisel 2022  USA RCT | Adults (18-70) with LBP that were likely to be discharged home, able to communicate in English  *did not specify if leg pain was present or serious spinal conditions  Excluded: patients in police custody, pregnant, under the influence of drugs or alcohol, suicidal or homicidal, mentally or cognitively unstable, unable to take opioids or NSAIDs, those who had taken opioid meds in the preceding 30 days, patients who exhibited drug-seeking behaviour | IG (n = 433): personalised opioid risk communicated via opioid risk tool visual aid and video narratives   CG (n = 434): generalised opioid risk information (information sheet including benefits, side effects, and risks of various analgesic options including opioids) | **Patient-focused:** *Train and educate* - opioid risk tool to communicate risk information to patient  *Engage consumers* - narratives used included speakers with varying gender, age and racial backgrounds and tested through iterative feedback process that included patient investigators and patient advisors; outcomes chosen were meaningful and important to patients and identified with patient investigators and a community and patient family engagement team; patients could choose which narrative they wanted to watch (shared-decision making)  - used patient investigators and developed a community and patient family engagement team  *Adapt and tailor to context* - patients perspectives on opioid prescribing were identified to inform the intervention (barriers/facilitators)   *Use evaluative and iterative strategies* - tool was developed and tested using an iterative process   **Clinician-focused:**  *Adapt and tailor to context* - clinician perspectives on opioid prescribing were identified to inform the intervention (barriers/facilitators)   *Engage consumers* - outcomes chosen were relevant to clinicians  *Stakeholder interrelationships* - outcomes relevant to health policy decision-makers   *Support clinicians* - opioid risk tool provided them with an objective measure of patients likely risk of misuse | **Patient:**  NPRS (during ED)  Analgesia preference (opioids)  Satisfaction with pain treatment (Mean - 1 day post)   Alignment patient preference with clinician prescription  **Quality of care:**  Opioids given in ED (n/%)  **Health service:** LOS (hours) | **Patient:**  IG 6.5 (SD 2.2) v CG 6.8 (SD 2.2)  IG 25.9% v CG 33%  Difference -7.00, 95% CI -13.1 to -1.0  IG 7.25 v CG 6.57  Difference 0.69, 95% CI 0.08 to 1.29  IG 70.1% v CG 65.9%  Difference 4.2, 95%  CI -2.0 to 10.4  **Quality of care:** IG 23.1% v CG 28.8%  OR 0.74, 95%  CI 0.55 to 1.01  **Health service:** IG 3.83 v CG 4.08  MD -0.25, 95%  CI -0.36 to -0.14 | Good quality (DB 75%) Probabilistic risk tool + narrative was more effective than general risk information in decreasing preference for opioids at discharge, satisfaction with pain treatment and shared decision-making. There was a reduction in the odds of receiving an opioid in the IG with uncertainty in the effect size estimate. Opioid risk tool patients spent 25 minutes less time in ED than patients who received general risk information |
| Min 2017  Canada  Before-after observational study | All visits with a diagnosis of acute LBP (<2 weeks) including serious spinal pathology and radicular back pain | IG (n = NR): after implementation of a point-of-care checklist of accepted "red flags" for LBP embedded into computerised order entry form for lumbar imaging   CG (n = NR): before implementation of an electronic CDS tool (usual care) | **Patient-focused:**  *Train and educate* - education summary provided to those patients where imaging was not indicated outlining why, options to manage their symptoms and when to seek medical attention (including Choosing Wisely pamphlet)  **Clinician-focused:**  *Support clinicians* - clinical decision support and reminder system embed into computerised request form  *Train and educate* - 2 months of education and communication to clinicians prior to process change   *Stakeholder interrelationships* - tool designed by physicians, radiologists and family physicians (early engagement of stakeholders)   **Health service-focused:**  *Systems-based* - change to order set or documentation requirements with point of care checklist and computerised order entry form limiting indications for approval of lumbar imaging | **Quality of care:**  Proportion imaging (imaging/total LBP visits)  Imaging within 30 days of being discharged | **Quality of care:** IG 17% v CG 22% p = 0.0002  IG 2.2% outpatients, 6.9% ED representation v CG 2.3% outpatients, 8.2% ED representation | Fair quality (DB 52%) After implementing a red flag checklist in the order entry form for lumbar imaging, study observed a 22% relative decrease in the median rate of imaging of LBP patients in the ED. In addition to the overall imaging rate for LBP, individual physician image ordering rates also decreased from 24% to 20% and the median imaging rate among individual physicians decreased from 23% to 20% |
| Peters 2022  Belgium Before-after observational study | All visits to the ED for a lumbar spine-related problem  *did not specify if leg pain was present or serious spinal conditions | IG (n = NR): after implementation of a new LBP protocol based on evidence-based guidelines and in keeping with organisational aspects of the ED and hospital   CG (n = NR): before implementation of protocol (usual care) | **Patient-focused:**  *Train and educate* - information brochures  **Clinician-focused:** *Stakeholder interrelationships* - GP referral and communication links, GP hotline to timely refer patients entering the subacute stage   *Train and educate* - face-to-face education sessions initially then replaced with compulsory eLearning; protocol available on hospital intranet; posters; GP symposium/education sessions  *Adapt and tailor to context* - protocol developed by ED clinicians in the context of the local ED environment   *Use evaluative and iterative strategies* - automated feedback to clinicians   **Health service-focused:**  *Systems-based* - creation of a new clinical team with task shifting from trauma surgeon to emergency physician; new clinical pathway with structured intake of patients attending ED with LBP; change to order set or documentation requirements with new LBP proforma; neurosurgery/orthopaedics on-call system   *Stakeholder interrelationships* - multidisciplinary team of specialists (physical medicine, rehabilitation specialists, neurosurgeon, orthopaedic surgeon, physiotherapist and care program delivery manager) | **Quality of care:**  Imaging rates | **Quality of care:** IG 14-14.6% CT use and 12.7-13.5% Xray use v CG CT and Xray use > 25%  Absolute reduction of around 10% | Poor quality (DB 42%)  Observed a significant decrease of both percentages from over 25% before implementation of the new protocol to 14-14.6% for CT scan use and 12.7-13.5% for Xray use after introduction of a compulsory eLearning package |
| Sapadin 2022  USA Before-after observational study | Adults (≥21 years) presenting to the ED who received either a thoracic or lumbar Xray (did not exclude serious pathology)  *did not specify if leg pain was present | IG (n = NR): after implementation of a multifaceted quality improvement intervention to improve the appropriateness of lumbar imaging   CG (n = NR): before quality improvement intervention being implemented (usual care) | **Patient-focused:**  *Train and educate* - brochures in waiting room, posters  **Clinician-focused:**  *Train and educate* - physician and provider education   *Support clinicians* - clinical decision support tool (best practice advisory card)   *Evaluative and iterative strategies* - audit and feedback targeting 5 highest users of Xray  **Health service-focused:**  *Systems-based* - change to order set or documentation requirements by altering electronic imaging requesting process | **Quality of care:**  % of appropriately ordered radiographs  Absolute number of radiographs | **Quality of care:** IG 53.2% v CG 5.8%  Absolute increase in appropriateness of 47.4%  IG 47/month v CG 90/month Absolute reduction in imaging of 52% | Poor quality (DB 46%) A multi-component quality improvement intervention led by a clinical champion resulted in a reduction in the number of radiographs ordered to evaluate LBP and an increase in the proportion of those ordered that were appropriate (total number decreased by almost 44% and of those that were ordered, the proportion that were appropriate increased by 10-fold) |
| Tacy 2017  USA  Before-after observational study | Adults (18-50) with chronic LBP, exacerbation of chronic LBP, or other related diagnoses according to back pain codes  *did not specify if leg pain was present   Excluded:  Serious spinal conditions, pregnancy, neurological compromise | IG (n = 89): participants seen in pilot period after implementation of protocol   CG (n = 46): participants seen in prelaunch period immediately preceding the pilot | **Patient-focused:**  *Train and educate* - LBP resources including discharge instructions   **Clinician-focused:**  *Train and educate* - education to ED providers, nurses, and staff; guidelines available in both electronic and hard copies in multiple areas   *Engage consumers* - nurse consultant project champions   *Adapt and tailor to context* - piloted and iterated protocol with process feedback to staff   **Health Service-focused:**  *Use evaluative and iterative strategies* - performed baseline needs evaluation  *Stakeholder interrelationships* - fast track outpatient referrals available; interprofessional collaboration between the ED, outpatient physiotherapy department, and spine centre  *Systems-based* - change to order set or documentation requirements with alterations to electronic medical record | **Patient:**  Pain reduction at discharge (>20% reduction)   **Quality of care:**  Prescriptions in line with guidelines  Back exercise instructions are given at discharge  Referral to specialty   **Implementation:**  Guideline use | **Patient:** IG 45% v CG 37.4%  **Quality of care:**  IG 90% v CG 82.6%  IG 45% v CG 37%  IG 30% v CG 23.9%   **Implementation:** IG 64% v CG 50% | Poor quality (DB 43%) More providers used the guidelines post-launch of the protocol. 90% of patients were discharged with guideline-based prescriptions after the launch of the protocol. Further work is needed on discharge instructions with only 45% of patients discharged with guideline-based advice. Patients were satisfied |

IG: intervention group; NR: not reported; CG: comparison group; OR: odds ratio; DB: Downs and Black; HIE: health information exchange; CDS: clinical decision support; SSU: short stay unit; ODI: Oswestry Disability Index; PROMIS-PI: Patient-Reported Outcome Measurement Information System; PT: physical therapy; NPRS: Numerical Pain Rating Scale; SD: standard deviation; NEAT: National Emergency Access Target; NSAIDs: non-steroidal anti-inflammatories; GP: General Practitioner

**TABLE S3:** Implementation strategies with cluster labels

| **Implementation strategy** | **Frequency of use across studies** | **Strategy label** |  |
| --- | --- | --- | --- |
|  |  |  |  |
| **Patient-focused strategies** | | |  |
| LBP pamphlets, handouts, or posters (14, 17, 19, 21, 38-40, 52) | 9 | Train and educate stakeholders |  |
| Opioid risk communication tool (53) | 1 | Train and educate stakeholders |  |
| Patient cost-sharing (55) | 1 | Financial |  |
| Behavioural cues (framing, cues to action, status quo) (17) | 1 | Engage consumers |  |
| Shared decision-making (patient could choose intervention) (53) | 1 | Engage consumers |  |
| Culturally and linguistically diverse patient opioid risk narratives (53) | 1 | Engage consumers |  |
| Patient investigators and engagement team (53) | 1 | Engage consumers |  |
| Patient-relevant outcomes (53) | 1 | Engage consumers |  |
| Community and patient family engagement team (53) | 1 | Engage consumers |  |
| Barriers and facilitators (patient perspectives) (53) | 1 | Use evaluative and iterative strategies |  |
| Intervention developed iteratively (53) | 1 | Use evaluative and iterative strategies |  |
| **n = 11 strategies** | 19 |  |  |
| **Clinician-focused strategies** | | |  |
| Guidelines only (35, 37, 50) | 3 | No active strategies used |  |
| Education seminars (14, 19, 21, 22, 34, 36, 38-40, 51) | 10 | Train and educate stakeholders |  |
| Education materials (hard copies of intervention or guidelines, departmental posters, videos) (14, 19, 36, 38, 40) | 8 | Train and educate stakeholders |  |
| Compulsory eLearning (19) | 1 | Train and educate stakeholders |  |
| Audit and feedback (14, 19, 21, 38) | 4 | Use evaluative and iterative strategies |  |
| Intervention developed iteratively (40) | 1 | Use evaluative and iterative strategies |  |
| Barriers and facilitators (clinician perspectives) (53) | 1 | Use evaluative and iterative strategies |  |
| New clinical assessment proforma with CDS (34, 38) | 2 | Support Clinicians |  |
| Automated printing of patient discharge paperwork (51, 54) | 2 | Support Clinicians |  |
| Clinical decision support (hard copy) (14, 21, 34, 38) | 4 | Support Clinicians |  |
| Physiotherapy phone line (34) | 1 | Support Clinicians |  |
| Non-opioid alternative treatment options (heat wraps) (14) | 1 | Support Clinicians |  |
| Website (14) | 1 | Support Clinicians |  |
| Access to external health information (48) | 1 | Support Clinicians |  |
| Clinician reminders (39) | 1 | Support clinicians |  |
| Clinician-relevant outcomes (39) | 1 | Engage consumers |  |
| Email from ED leadership (17) | 1 | Engage consumers |  |
| Clinical champions (38, 40) | 2 | Engage consumers |  |
| Outcomes relevant to health policy and decision-makers (53) | 1 | Stakeholder interrelationships |  |
| GP hotline and community links (19) | 1 | Stakeholder interrelationships |  |
| Clinical mentorship from ED or external specialties (19) | 1 | Stakeholder interrelationships |  |
| Key stakeholders developed intervention (39) | 1 | Stakeholder interrelationships |  |
| Intervention developed by ED clinicians (19) | 1 | Adapt and tailor to context |  |
| **n = 23 strategies** | 50 |  |  |
| **Health service-focused strategies** | | |  |
| New clinical pathway (19, 34, 42-47, 52) | 8 | Systems-based |  |
| Change to order set or documentation requirements with CDS (21, 39-41, 49, 51, 54) | 7 | Systems-based |  |
| Computerised medical record with CDS and automation (51, 54) | 2 | Systems-based |  |
| Task shifting (19, 22) | 2 | Systems-based |  |
| On-call system (19) | 1 | Support clinicians |  |
| Multidisciplinary LBP care team (19) | 1 | Stakeholder interrelationships |  |
| Fast-track outpatient referrals (14, 38, 40, 44) | 4 | Stakeholder interrelationships |  |
| Interprofessional collaboration (40) | 1 | Stakeholder interrelationships |  |
| Stakeholder agreement on new processes (38) | 1 | Stakeholder interrelationships |  |
| Baseline needs analysis (40) | 1 | Use evaluative and iterative strategies |  |
| **n = 10 strategies** | 29 |  |  |
| **TOTAL = 44 STRATEGIES** | **97** |  |  |
|  | | |  |
|  |  |  |  |

**TABLE S4:** Cochrane Risk of Bias assessment of RCTs

|  | Sequence Generation | Allocation concealment | Blinding of participants and personnel  (performance bias) | Blinding of outcome assessment  (detection bias) | Incomplete outcome data  (attrition bias) | Selective reporting  (reporting bias) | Other bias |  |
| --- | --- | --- | --- | --- | --- | --- | --- | --- |
| Coombs *et al 2021* | *Low* The randomisation schedule, based on a computer-generated random sequence. Baseline and intervention group characteristics were similar suggesting randomisation was adequate | *Low* The timing of commencement of the intervention was concealed from clusters and site investigators until 2 months before implementation | *High*  It was not possible to blind the clinician participants, patients, or investigators. Potential for hospital staff to cross one or more intervention sites. | *Low*  Primary outcome measures were extracted from electronic medical records by the data provider of each hospital independent from the research team   Assessors of patient-reported outcomes were blinded to group allocation | *Low* Small percentages of missing data for some variables >5%. Low percentage missing data for primary outcome | *Low* Balanced reporting of results that overall were not highly favouring of the intervention | *Low* Diagnostic coding methods  Compliance with intervention Participants recruited over different time periods |  |
| Lau *et al 2008* | *Low* Randomisation was generated from a random numbers table. A block size of 10 was used to ensure that comparison groups would be of approximately the same size and the allocation sequence was sealed in sequentially numbered identical envelopes | *Low*  The therapists responsible for screening the acute LBP patients in ED ensured that the envelopes were opened sequentially, and that group allocation was only revealed to the treating therapists once recruitment and baseline data collection were completed | *Unclear* The study involved unequal amounts of intervention between the 2 groups. The patients receiving the intervention may have experienced more "demand" to report success. The therapists involved would have known which group the patient was in and may have provided biased emphasis on outcomes and reporting | *Low*  Assessors of patient-reported outcomes were blinded to group allocation. | *Low*  Analysis was by intention-to-treat in that all randomised participants with follow-up data available were analysed. No attempt was made to impute values of missing data.  Attrition and follow up rates were reported (90%). Patients lost to follow up numbers were provided and included in the analysis (reasons were given as to why they were not compliant with follow up in flow chart). These were similar between groups.  Missing outcome data for Back Performance Scale (3 and 6 month follow up in both groups) however this does not affect ED outcomes | *Low* | *Unclear* Both groups received "standard medical intervention of pain-relieving drugs" however exactly what standard medical intervention they received is unclear. As such the outcomes they received may be due to other medical treatment that the participants may have had and not the intervention.  Three participants in the intervention group had to be admitted to the hospital however their data was included in the analysis. |  |
| Meisel 2022 | *Low* Electronic consent and simple unblinded randomisation within strata and hospital centres were conducted automatically during enrolment via computer-generated random numbers. Baseline and intervention group characteristics were similar suggesting randomisation was adequate | *High* No information on allocation concealment provided | *Unclear* Patients would not have been aware of grouping for study  Clinicians likely would have known the difference between interventions, but it is unclear whether they knew the outcomes of the study | *Low* In protocol paper it states that they increased sample size during the study "without unblinding the data" so a level of blinding can be inferred however this is not explicit in the main paper. Main outcomes were not open to interpretation | *Low* Lost to follow up numbers are reported in protocol paper with reasons given. All eligible data was analysed (no data were excluded from analysis) | *Low* Balanced reporting of results that overall were not highly favouring of the intervention. | *Unclear* Intervention compliance Adequate adjustment for confounding Unclear source population |  |

**Figure S1:** Distribution of true effects (mean effect size with 95% prediction interval)

**Table S5:** summary of findings for subgroup analyses – lumbar imaging

| **Summary of findings - exploratory subgroup analyses** | | | | |
| --- | --- | --- | --- | --- |
| Imaging subgroup analyses | | | | |
| **Moderator variable** | Studies | Participants | I^2^, % | OR (95% CI) |
| Intervention target |  |  |  |  |
| Patient | 1 | 337 | 0 | 0.83 (0.32, 2.16) |
| Clinician | 5 | 7850 | 81.71 | 0.89 (0.59, 1.33) |
| Health service | 4 | 1617 | 14.23 | 0.75 (0.43, 1.31) |
|  | Q-value = 0.24 P-value = 0.89 (no difference between groups) | | | |
| Risk of Bias |  |  |  |  |
| Low | 2 | 4962 | 0 | 0.80 (0.47, 1.37) |
| Moderate | 5 | 3043 | 53.84 | 1.05 (0.73, 1.52) |
| High | 3 | 1799 | 72.14 | 0.68 (0.44, 1.04) |
|  | Q-value = 2.47 P-value = 0.29 (no difference between groups) | | | |
| Case definition |  |  |  |  |
| LBP only | 3 | 1916 | 59.8 | 0.99 (0.56, 1.73) |
| Includes participants with radicular symptoms | 4 | 6839 | 9.35 | 0.79 (0.46, 1.36) |
| Includes participants with radicular symptoms and serious/specific pathologies | 2 | 712 | 92.08 | 0.72 (0.37, 1.40) |
| Includes participants with radicular symptoms, serious/specific pathologies and potential non-MSK diagnoses | 1 | 337 |  | 0.83 (0.31, 2.20) |
|  | Q-value = 0.56 P-value = 0.91 (no difference between groups) | | | |
| Codes |  |  |  |  |
| Triage | 6 | 3690 | 75.71 | 0.81 (0.56, 1.17) |
| Discharge | 4 | 6114 | 48.63 | 0.90 (0.55, 1.50) |
|  | Q-value = 0.12 P-value = 0.73 (no difference between groups) | | | |
| Baseline imaging rates |  |  |  |  |
| High | 3 | 775 | 63.02 | 0.60 (0.39, 0.93) |
| Low | 7 | 9029 | 45.43 | 0.99 (0.76, 1.31) |
|  | **Q-value = 3.70 P-value = 0.05 (greater effect in high imaging studies)** | | | |
| Imaging modality |  |  |  |  |
| Any imaging | 6 | 6552 | 26.96 | 0.85 (0.56, 1.28) |
| Xray only | 4 | 3252 | 84.71 | 0.83 (0.54, 1.29) |
|  | Q-value = 0.003 P-value = 0.96 (no difference between groups) | | | |
| Included systems-based changes |  |  |  |  |
| Yes | 5 | 1929 | 58.8 | 0.65 (0.45, 0.94) |
| No | 5 | 7875 | 41.1 | 1.04 (0.77, 1.41) |
|  | **Q-value = 3.85 P-value = 0.05 (greater effect with systems-based changes)** | | | |

**Figure S2**: Forest plot of comparison. Outcome: non-opioids

**Figure S3**: Forest plot of comparison. Outcome: ED LOS

**Figure S4:** Distribution of true effects ED LOS (mean effect size with 95% prediction interval)

**Table S6:** GRADE assessment

| **Certainty assessment** | | | | | | | **№ of patients** | | **Effect** | | **Certainty** | **Importance** |
| --- | --- | --- | --- | --- | --- | --- | --- | --- | --- | --- | --- | --- |
| **№ of studies** | **Study design** | **Risk of bias** | **Inconsistency** | **Indirectness** | **Imprecision** | **Other considerations** | **Intervention** | **Control** | **Relative (95% CI)** | **Absolute (95% CI)** |  |  |
| **Imaging (assessed with: Proportion of people with LBP that received lumbar imaging in ED)** | | | | | | | | | | | | |
| 10 | observational studies^a^ | serious^b^ | serious^c^ | not serious | serious^d^ | none | 785/3547 (22.1%) | 1320/6257 (21.1%) | **OR 0.87** (0.69 to 1.09) | **2 fewer per 100** (from 6 fewer to 1 more) | ⨁◯◯◯ Very low |  |
| **Opioids** | | | | | | | | | | | | |
| 6 | observational studies^e^ | not serious^f^ | not serious^g^ | not serious | not serious | none^h^ | 1136/2972 (38.2%) | 2289/4389 (52.2%) | **OR 0.69** (0.63 to 0.77) | **9 fewer per 100** (from 11 fewer to 7 fewer) | ⨁⨁◯◯ Low |  |
| **Non-opioid analgesic** | | | | | | | | | | | | |
| 3 | observational studies^i^ | not serious^j^ | not serious^k^ | not serious | not serious | none^l^ | 1286/2056 (62.5%) | 2238/3604 (62.1%) | **OR 1.33** (1.18 to 1.50) | **6 more per 100** (from 4 more to 9 more) | ⨁⨁◯◯ Low |  |
| **Length of stay (assessed with: hours)** | | | | | | | | | | | | |
| 7 | observational studies | serious^m^ | very serious^n^ | not serious | serious^o^ | none | MD 0.72 lower (1.3 to 0.13 lower)  Intervention = 2583, control = 5382 participants 2 RCTs (1 cRCT) with 5492 participants 1 study showed a reduction of approximately 25mins ED LOS with the other study demonstrating no difference in LOS between groups 4 observational cohort studies 1 observational before-after studies 2473 participants 1 study reported a reduction in LOS of 5 hours in the intervention group with another study reporting a redution of 2 hours and a third study reporting a reduction of 50 mins. Two studies reported no significant difference in LOS between groups. | | | | ⨁◯◯◯ Very low |  |
| **Admission** | | | | | | | | | | | | |
| 3 | observational studies^p^ | not serious | very serious^q^ | not serious | not serious | none | 445/1872 (23.8%) | 1423/4662 (30.5%) | **OR 0.74** (0.65 to 0.83) | **6 fewer per 100** (from 8 fewer to 4 fewer) | ⨁◯◯◯ Very low |  |
